# Comprehensive Bioinformatics Analysis of the Biodiversity of Lsm Proteins in the *Archaea* Domain

**DOI:** 10.3390/microorganisms11051196

**Published:** 2023-05-03

**Authors:** Gloria Payá, Vanesa Bautista, Mónica Camacho, Julia Esclapez, María-José Bonete

**Affiliations:** Department of Biochemistry and Molecular Biology and Soil Science and Agricultural Chemistry, Faculty of Science, University of Alicante, Ap 99, 03080 Alicante, Spain; gloria.paya@ua.es (G.P.); vanesa.bautista@ua.es (V.B.); camacho@ua.es (M.C.); julia.esclapez@ua.es (J.E.)

**Keywords:** Lsm, archaea, RNA metabolism, bioinformatics analysis

## Abstract

The Sm protein superfamily includes Sm, like-Sm (Lsm), and Hfq proteins. Sm and Lsm proteins are found in the *Eukarya* and *Archaea* domains, respectively, while Hfq proteins exist in the *Bacteria* domain. Even though Sm and Hfq proteins have been extensively studied, archaeal Lsm proteins still require further exploration. In this work, different bioinformatics tools are used to understand the diversity and distribution of 168 Lsm proteins in 109 archaeal species to increase the global understanding of these proteins. All 109 archaeal species analyzed encode one to three Lsm proteins in their genome. Lsm proteins can be classified into two groups based on molecular weight. Regarding the gene environment of lsm genes, many of these genes are located adjacent to transcriptional regulators of the Lrp/AsnC and MarR families, RNA-binding proteins, and ribosomal protein L37e. Notably, only proteins from species of the class Halobacteria conserved the internal and external residues of the RNA-binding site identified in *Pyrococcus abyssi*, despite belonging to different taxonomic orders. In most species, the Lsm genes show associations with 11 genes: *rpl7ae, rpl37e, fusA, flpA, purF, rrp4, rrp41, hel308, rpoD, rpoH*, and *rpoN*. We propose that most archaeal Lsm proteins are related to the RNA metabolism, and the larger Lsm proteins could perform different functions and/or act through other mechanisms of action.

## 1. Introduction

The Sm protein superfamily includes Sm, like-Sm (Lsm), and Hfq proteins, which are all involved in the RNA metabolism [1,2,3]. Sm and Lsm proteins are found in *Eukarya* and *Archaea* domains, respectively, while Hfq proteins exist in the *Bacteria* domain and one archaeon species, *Methanocaldococcus jannaschii* [2,3,4,5]. Sm and Lsm proteins differ at the amino acid sequence level from Hfq; however, they show striking similarities in their tertiary and quaternary structure levels [3,6,7]. 

This Sm protein superfamily has a bipartite sequence known as the Sm motif, which consists of two segments, the Sm1 and Sm2 motifs, separated by a region of variable amino acid sequence and length [7]. The secondary structure of this protein superfamily consists of a short α-helix (two to four turns) at the N-terminal end and five β-strands, identified from the N-terminal to the C-terminal end as β1, β2, β3, β4, and β5. The Sm1 motif corresponds to the β1, β2, and β3 strands, and the Sm2 motif corresponds to the β4 and β5 strands [8].

Eukaryotic Sm proteins are part of spliceosomes and remove introns from pre-mRNAs [9]. Moreover, numerous studies have shown that eukaryotic Sm proteins function as molecular scaffolds for RNP assembly and are involved in mRNA degradation, folding, stabilization, splicing, telomere maintenance, and histone maturation [7]. In addition, archaeal Lsm proteins were discovered by searching for homologous sequences in databases [10]. 

These proteins were not necessarily expected in archaea due to the absence of introns in their genes and their primitive RNA processing machinery [11]. However, crystallographic studies showed that the architecture of the Sm core motif and the RNA-binding site of these proteins are conserved in both eukaryotes and archaea, including the Lsm subfamily [8,12,13,14].

On the other hand, the Hfq protein was discovered for replicating the RNA phage Qβ more than 50 years ago [15,16,17]. However, it is now known to have many cellular functions [6]. For example, one of the identified functions of Hfq is regulating gene expression through its interaction with sRNAs. Hfq acts as a chaperone for regulatory sRNAs and their target mRNAs, thus, facilitating their interaction [18].

Although the physiological functions of archaeal Lsm proteins are unknown, several protein structures have been solved in *Archaeoglobus fulgidus, Methanobacterium thermoautotrophicum, Pyrobaculum aerophilum,* and *Halobacterium salinarum* R1 [8,12,13,14,19]. In addition, *A. fulgidus* Lsm proteins interact with each other and with RNase P, such as eukaryotic Sm [8]. Other studies showed that the archaeal Lsm protein could bind sRNAs, suggesting a similar function in regulating sRNAs by Hfq in Bacteria [20,21]. 

Furthermore, Lsm1 and Lsm2 from *Sulfolobus solfataricus* copurified with several proteins involved in RNA processing/modification and translation and with exosome components involved in mRNA degradation [22]. Deletion mutants of the *lsm* gene have been successfully generated in two species of haloarchaea, specifically in *Haloferax volcanii* [20] and *Haloferax mediterranei* [23]. The *lsm* deletion mutant of *Hfx. volcanii* has been characterized on different carbon sources showing differences in growth. 

In addition, the expression was analyzed under different growth conditions, and it was observed that, under certain conditions, it cotranscribes with the overlapping gene encoding the ribosomal protein L37r [20]. In *Hfx. mediterranei,* the *lsm* deletion mutant, and the Sm1 motif deletion mutant, in which the Sm2 motif remained intact, were generated and characterized. Comparison of the *lsm* deletion mutant, the Sm1 deletion mutant, and the parental strain HM26 under standard growth and stress conditions revealed differences in growth. 

These results indicate that the Lsm protein is involved in standard and stress growth conditions (low/high salinity, low/high temperature, heat shock, oxidative stress, and ethanol stress). Furthermore, expression of the *lsm* and *rpl37e* genes was constitutive, and the cotranscription of both occurs at suboptimal salt concentrations and temperatures [23]. The role of Lsm in *Archaea* is unknown, and more work is needed to elucidate if Lsm proteins, e.g., act as chaperones that facilitate the folding of sRNAs as in bacteria and/or act as structural scaffolds for the assembly of RNPs as in eukaryotes [11].

Additionally, the analysis of the amino acid sequences of proteins provides insight into the general characteristics and biochemical properties they share [24]. On the other hand, the construction of phylogenetic trees shows the relationship between sequences in a graphical representation. Moreover, describing a protein function requires knowing all the molecules with which the protein of interest can establish an association. From a functional perspective, the association can refer to the physical binding of proteins by ligands or an indirect interaction, such as participation in the same metabolic pathway or process [25]. 

Finally, in determining the biological functions of a protein, it is crucial to identify the presence of conserved elements associated with specific functions, which constitute the different motifs and domains. Residues directly or indirectly involved in a function can be grouped into these elements as a fingerprint, conserved in different proteins that share a given function [26]. In this work, various bioinformatics tools are used to understand the diversity and distribution of these proteins in the *Archaea* domain and, thus, increase the global understanding of Lsm proteins.

## 2. Materials and Methods

### 2.1. Selection Criteria for Lsm Proteins from the Archaea Domain

At least one species from each genus of the *Archaea* domain was selected, analyzing a total of 109 species to obtain an overview of the Lsm proteins. The UniProt database (http://www.uniprot.org/) (accessed on 15 January 2022) [27] was then manually retrieved for each species’ presence of Lsm proteins. All Lsm protein sequences (163 proteins from 109 species) were obtained in FASTA format for subsequent analyses.

### 2.2. Prediction of Physicochemical Properties

Amino acid sequences are essential for understanding a protein’s physicochemical, structural, and functional properties. For each of the 163 Lsm proteins, the Expasy ProtParam tool (https://web.expasy.org/protparam/) (accessed on 22 January 2022) [24] was used to calculate the physicochemical properties, such as the molecular weight (MW), isoelectric point (IP), extinction coefficient (EC) [28], instability index (II) [29], aliphatic index (AI) [30], grand average of hydropathy (GRAVY) [31], number of positively charged residues, and number of negatively charged residues.

### 2.3. Gene-Environment Analysis

For the identification and comparison of the gene environment of the *lsm* genes of different archaeal species, the Gene tool of the NCBI database (https://www.ncbi.nlm.nih.gov/gene/) (accessed on 7 February 2022) was used to analyze the environment of 76 Lsm proteins from 48 archaeal genomes.

In most species of the class Halobacteria, the *lsm* and *rpl37e* genes overlap (11 proteins from the Halobacteria class and 65 from other classes of the 76 Lsm proteins from 48 previously analyzed archaeal genomes). Thus, it was decided to analyze this aspect further by studying a more significant number of species of this class (in particular, 55 species and another 25 species of other classes of the phylum Euryarcheaota) to confirm whether this characteristic is widely distributed within the class Halobacteria. Therefore, the number of overlapping nucleotides and the distance between both genes were analyzed.

### 2.4. Phylogenetic Analysis of the Lsm Proteins of the Archaea Domain

The phylogenetic analysis of the Lsm proteins of the *Archaea* domain was performed using the Molecular Evolutionary Genetics Analysis 11 (MEGA 11) software [32]. Since most Lsm proteins belong to a group of smaller proteins (54–105 residues), it was decided to perform the phylogenetic analysis of this group of 137 proteins. 

First, a multiple alignment of the 137 protein sequences was performed with Clustal W (https://www.ebi.ac.uk/Tools/msa/clustalw2/) (accessed on 1 March 2022). Then, the best substitution model for protein sequences among the different fitted models was obtained using the Find Model tool of MEGA 11. Phylogenetic inference was performed using the maximum likelihood method. The phylogenetic tree was obtained automatically using the Neighbor Joining (NJ) algorithm with the Poisson model.

Once the phylogenetic tree was obtained, the protein–RNA, protein–protein interaction residues, and conserved structural motifs were analyzed using the NCBI Conserved Domains tool [33]. The secondary structure was analyzed using the Jpred 4 program [34] for each group obtained in the phylogenetic tree.

### 2.5. Multiple Alignments of Lsm Protein Sequences

Multiple sequence alignments were performed using Clustal Omega with default parameters [35] to visualize highly conserved sequence regions in Lsm proteins. Two different strategies were considered when clustering the different sequences. On the one hand, multiple sequence alignments were based on sequence length, where two groups were identified, larger proteins (139–164 residues) and smaller proteins (54–105 residues). On the other hand, alignments were performed based on the taxonomic class. The consensus sequences of the Lsm proteins were obtained based on the taxonomic order to which they belong, and a comparison of these sequences was performed using the MView tool (https://www.ebi.ac.uk/Tools/msa/mview/) (accessed on 1 March 2022).

### 2.6. Analysis of Protein–Protein Interaction Networks of Lsm Proteins

Gene–gene or protein–protein associations derived from experimental and bibliographic information were searched for the 163 selected proteins from 109 species of the *Archaea* domain by the STRING bioinformatics tool [25], but only 74 proteins were deposited in this database.

## 3. Results and Discussion

### 3.1. Selected Lsm Proteins and Their Distribution in the Archaea Domain

The Lsm proteins of each species were manually retrieved from the UniProt database (http://www.uniprot.org/) (accessed on 15 January 2022) [27], obtaining a total of 163 proteins, which are encoded in a total of 109 different species (Table S1).

**Figure 1:**
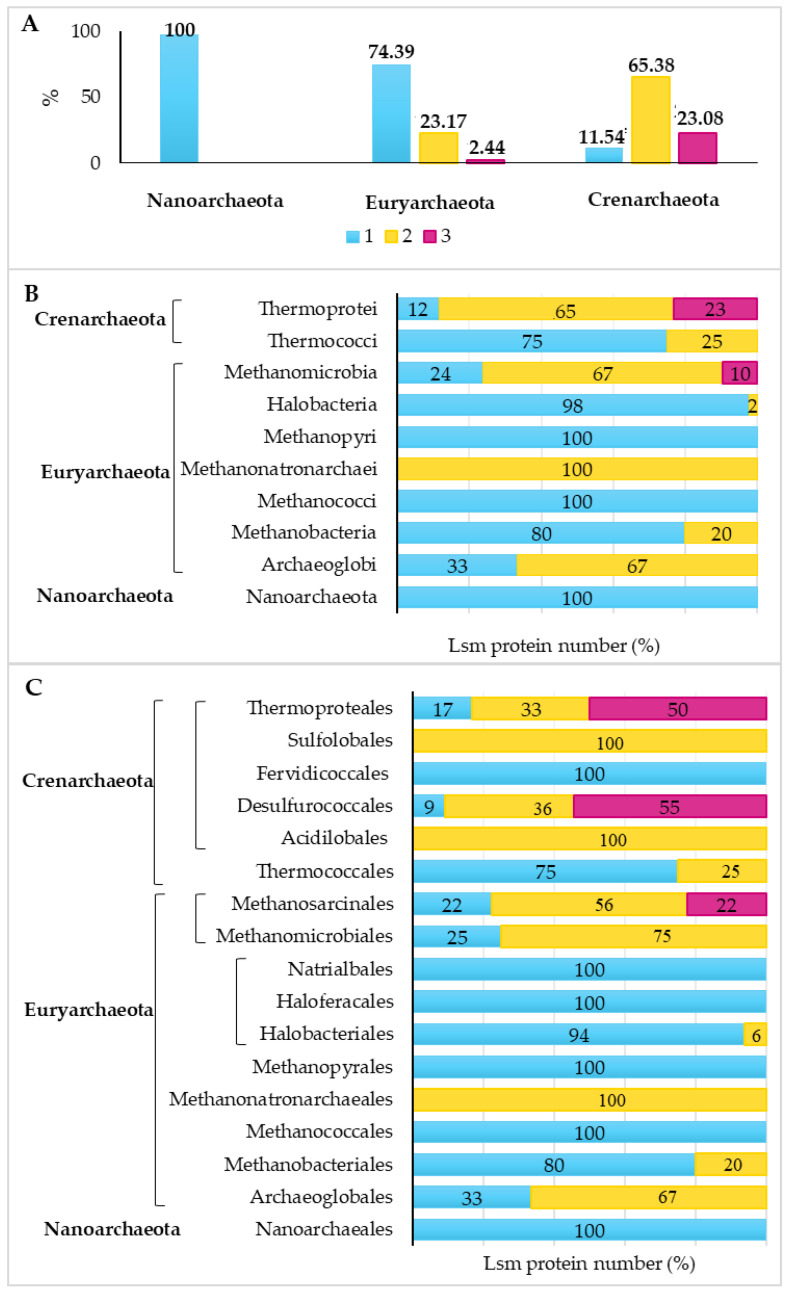
Distribution of the number of proteins according to phylum (**A**), class (**B**), and order (**C**) in the *Archaea* domain.

In these analyzed species, one to three *lsm* genes are encoded per genome. Most species of the phylum Crenarchaeota have two Lsm proteins encoded (65.38%), while most species of the phylum Euryarchaeota have one Lsm protein encoded in their genome (74.39%) (Figure 1A). The phylum Crenarchaeota presents a greater diversity in the number of Lsm proteins in each order (Figure 1C); all species of the order Fervidicoccales present one Lsm protein, while all species of the orders Sulfolobales and Acidilobales present two Lsm proteins. Interestingly, most species of the orders Thermoproteales and Desulfurococcales have three Lsm proteins, found in 50% and 55% of the analyzed species of these orders, respectively. 

As mentioned previously, species of the phylum Euryarchaeota mostly have one Lsm protein encoded in their genome (Figure 1A), except for most species of the classes Methanomicrobia, Methanonatroarchaeia, and Archaeoglobi, which have two Lsm proteins (Figure 1B). In addition, only in the class Methanomicrobia, there are species with three Lsm proteins (Figure 1B), specifically in the order Methanosarcinales (Figure 1C).

### 3.2. Prediction of Physicochemical Properties

**Figure 2:**
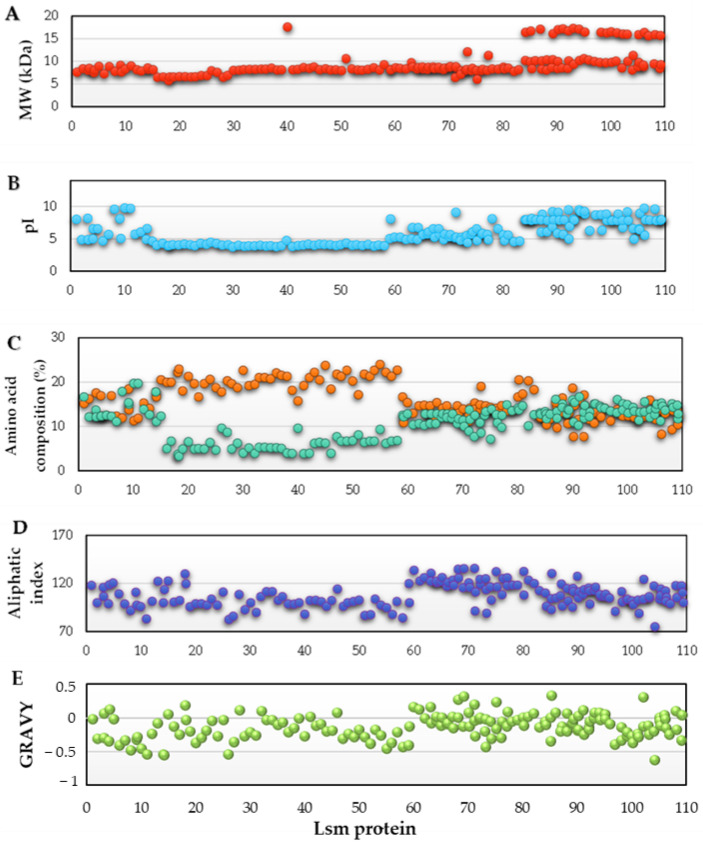
Physicochemical characteristics of the Lsm proteins of the *Archaea* domain. Molecular weight (**A**); pI (**B**); amino acid composition: percentage of negatively charged residues (Asp + Glu) (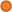) and percentage of positively charged residues (Arg + Lys) (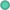) (**C**); aliphatic index (**D**); and GRAVY (**E**), for each species (represented on the X-axis and listed in Table S1).

The general characteristics and biochemical properties of 163 Lsm proteins were analyzed using Expasy’s ProtParam tool [24] (Figure 2 and Table S2).

Molecular weight (MW). There are two distinct groups of Lsm proteins: 5–12 kDa (54–105 residues) and 15–18 kDa (139–164 residues) (Figure 2A). Interestingly, the group of proteins with the highest MW is exclusively located in the phylum Crenarchaeota, except the Lsm protein from *Halobaculum gomorrense* (164 residues), which is found in the phylum Euryarchaeota.

Theoretical isoelectric point (pI). The pI of the proteins in our study is in a wide range of 3.75–9.75 (Figure 2B). The proteome of haloarchaea is characterized by being very acidic, so many of the proteins with a neutral or alkaline pI are usually membrane proteins or have specific functions, such as binding to nucleic acids (DNA or RNA), which are negatively charged [36]. Although Lsm proteins can bind RNA, all Lsm proteins belonging to the Halobacteria class have a narrow pI range (3.75–4.75). This narrow pI range is because species of the Halobacteria class are mainly found in hypersaline lakes and solar salt flats, so they are adapted to grow in salt concentrations above 2 M NaCl and, therefore, have a very acidic proteome close to pI 4.4 [36,37]. In contrast, most proteins with pI above 7.8 belong to the phylum Crenarchaeota. Therefore, the Lsm proteins have a pI close to the average pI of their respective proteomes.

Acid and basic amino acid composition. 

As expected, all archaeal species have a similar percentage of acidic and basic residues, except for species of the class Halobacteria (Figure 2C). As mentioned above, this class has an acidic pI (Figure 2B), corresponding to a higher percentage of negatively charged residues; this higher percentage of Glu +Asp residues is one of the different strategies to adapt proteins to work in environments with high salt concentrations [38,39].

Aliphatic index. The aliphatic index of a protein is the relative volume occupied by the aliphatic side chains: alanine, valine, isoleucine, and leucine. A high aliphatic index is an indicator of increased thermostability. For example, the aliphatic index of proteins from thermophilic bacteria is significantly higher than that of proteins from mesophilic species [30]. Most cytosolic enzymes have aliphatic indices around 80–100 [40] as do most of the Lsm proteins analyzed in this study (Figure 2D). In addition, Lsm proteins from the phylum Crenarchaeota have aliphatic indices above 100, as many thermophilic species are found in this phylum.

Grand average of hydropathy (GRAVY). The GRAVY value of a peptide or protein is calculated as the sum of all amino acids’ hydropathy values divided by the sequence’s number of residues. Polar residues have more negative values, while hydrophobic residues receive more positive values [31]. The GRAVY values for the Lsm proteins in this study were negative or null (typical of soluble proteins) for all except for some Lsm proteins of the phylum Crenarchaeota (Figure 2E).

### 3.3. Gene-Environment Analysis

The identification and comparison of the *lsm* gene environment of the different archaeal species were conducted using the Gene tool of the NCBI database (https://www.ncbi.nlm.nih.gov/gene/) (accessed on 7 February 2022), obtaining information on the environment of 76 *lsm* genes of a total of 48 archaeal species (Table S3). 

Many of the *lsm* genes are located adjacent to transcriptional regulators. The type of transcriptional regulators belongs to the Leucine-responsive regulatory protein/Asparagine-responsive regulatory protein (Lrp/AsnC) and MarR families, both transcriptional regulators with H-T-H (Helix-Turn-Helix) domains. In bacteria, transcriptional regulators of the Lrp/AsnC family are related to amino acid biosynthesis [41,42], while in archaea, they are considered global regulators in response to environmental changes. In the case of *Pyrococcus furiosus*, *Sulfolobus solfataricus*, *Methanocaldococcus jannaschii*, and *Halobacterium salinarum*, these regulators are highly versatile in their DNA-binding properties, response to effector molecules and molecular regulatory mechanisms [43,44,45,46,47,48,49]. 

In *Hfx. mediterranei* Lrp seems to be involved in a general response against stress factors [50] and mediates regulation in the stress response, especially under N-limiting conditions and in the presence of cobalt [51]. On the other hand, the MarR family regulators include a series of transcription factors that modulate genes in response to environmental signals by acting as sensors in changing environments. This family regulates the activity of genes involved in responses to different types of stress, virulence factors, the export of toxic compounds and antibiotics, and metabolic pathways [52,53].

Other genes commonly present in the gene environment of the Lsm protein are the ribosomal protein L37e and RNA-binding proteins. These RNA-binding proteins are characterized by the PUA domain, which is found in archaeal and eukaryotic enzymes involved in RNA modification and in bacterial and yeast glutamate kinases, in which the role of these enzymes in regulating the expression of other genes has been demonstrated [33]. 

The ribosomal protein L37e is found adjacent to the *lsm* gene in many archaea species. Its main function is stabilizing the interactions between the domains to maintain the structural integrity of the 50S ribosomal subunit. L37e is located within the large ribosomal subunit, specifically in the RNA-binding pocket, and is the protein that presents the highest percentage of its surface area (65%) to RNA interactions. L37e and L39e are hypothesized to bind to RNA 23S during the assembly of the 50S subunit [54]. 

Interestingly, in all species of the Halobacteria class analyzed, the *lsm* and *rpl37e* genes overlap in the same direction of transcription, indicating that both genes are cotranscribed. Furthermore, it has been shown that these genes are cotranscribed in species of *Haloferax* [20,23]. Very similar gene environments were found in *Hfx. mediterranei, Haloquadratum walsbyi* DSM16790, *Halohasta litchfieldiae,* and *Halorubrum lacusprofundi* ATCC49239. These have, upstream of the *lsm* gene, the gene encoding ribonuclease J, and, downstream, the gene encoding ribosomal protein L37e overlapping with *lsm*. In *Bacillus subtilis*, RNase J, which shares functional homologies with the *Escheriachia coli* RNase E, is involved in specific mRNA processing and global mRNA degradation [55]. 

Although the functions of ribonucleases in archaea are not yet as well understood as in the other domains of life, the available data suggest that these ribonucleases may have generalized functions in all three domains, namely, exoribonucleolytic degradation of mRNA in the 5′-3′ sense and sensitivity to the phosphorylation state of the 5′ end of a transcript [56]. This activity has been demonstrated in characterizing the RNase J of the hyperthermophilic Euryarchaeota *Pyrococcus abyssi* and *Thermococcus kodakaraensis* [57].

Exclusively in the phylum Crenarchaeota, we find, adjacent to the *lsm* gene, different genes, such as the N subunit of RNA polymerase; tRNA guanosine transglycosylase, responsible for tRNA modification; and methionine adenosyltransferase, whose biological functions include acting as a primary donor of methyl groups, as a precursor of polyamines and as a progenitor of 5′-deoxyadenosyl radical [58].

In most of the species of the class Halobacteria, the *lsm* and *rpl37e* genes overlap (Table S3), so it was decided to study this aspect further and to analyze a more significant number of species of this order, particularly 55 species, and another 25 species of other classes of the phylum Euryarcheaota, to confirm that this is a widely distributed characteristic within the class Halobacteria. The *lsm* and *rpl37e* genes overlap four nucleotides in all the species analyzed from Halobacteria and Thermoplasmata. In the rest of the species of other classes, these genes are not overlapping, although, in most cases, they are adjacent in the genome, with the following distance ranges: Archaeoglobi, 6–20 nucleotides; Methanobacteria, 58–112 nucleotides; Methanococci, 76–113 nucleotides; Methanomicrobia, 17–495 nucleotides; and Thermococci, 11–25 nucleotides (Table S4).

### 3.4. Multiple Alignments of Lsm Protein Sequences

**Figure 3:**
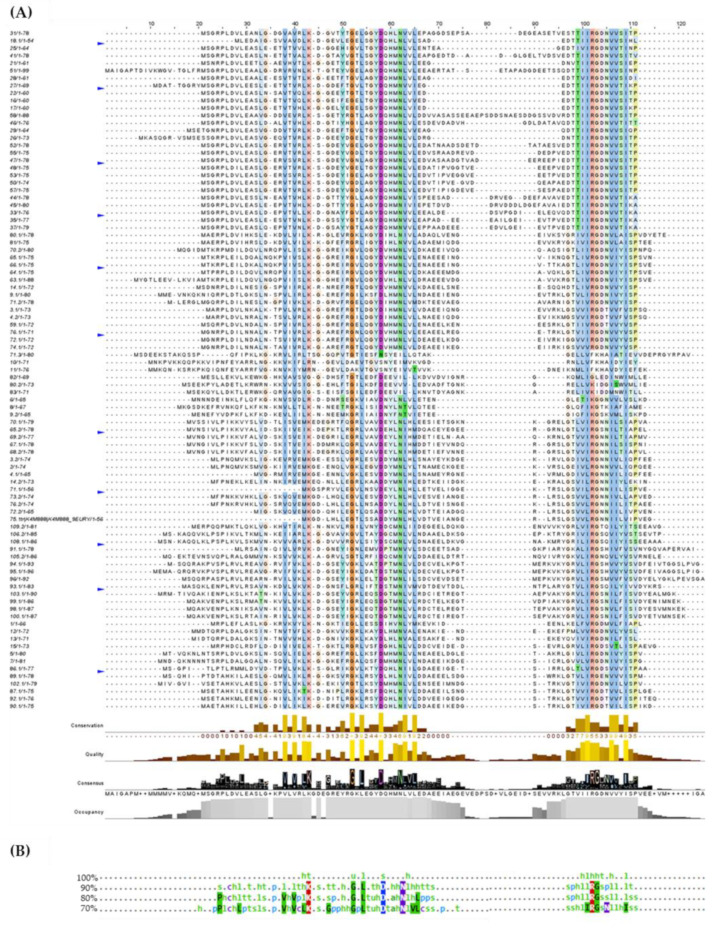
Alignment of the 137 protein sequences (54–105 residues in size) by Clustal Omega visualized with Jalview (**A**) and consensus sequences obtained by MView (**B**).

Multiple sequence alignments were based on sequence length in which two groups were identified: smaller proteins (54–105 residues) and larger proteins (139–164 residues). The proteins are highly conserved throughout the *Archaea* domain, specifically in the regions corresponding to the Sm1 and Sm2 motifs, with a region varying in size and residues between the two motifs (Figure 3A). The proposed function of the Lsm proteins is to facilitate RNA–RNA interactions; specifically, they are associated with uracil-rich RNA sequences. In *P. abyssi*, the general structure of the protein is a heptameric ring with a central cavity, such as the Sm proteins of eukaryotes. 

RNA molecules bind to the protein at two different sites: within the ring with three residues defining the uridine-binding pocket and on the surface of the α-helix located in the N-terminal region. The internal uracil-binding pocket is formed by residues His-37, Asn-39, and Arg-63. The uracil base establishes contacts with His-37 and Arg-63. The binding pocket is stabilized by a salt bridge between Arg-63 and Asp-65, forming an ionic interaction with Lys-22. In addition, the hydrogen bonds at Asp-35 and Asn-39 make this binding site specific for uridine [59]. Lys-22, Asp-35, Asn-39, and Arg-63 are highly conserved (>90% of the sequences analyzed) in the *Archaea* domain. On the other hand, the external RNA binding site residues in the α-helix are Arg-4, Asp-7, His-10, and Tyr-34 in *P. abyssi*, which do not appear to be conserved in the *Archaea* domain (Figure 3B).

**Figure 4:**
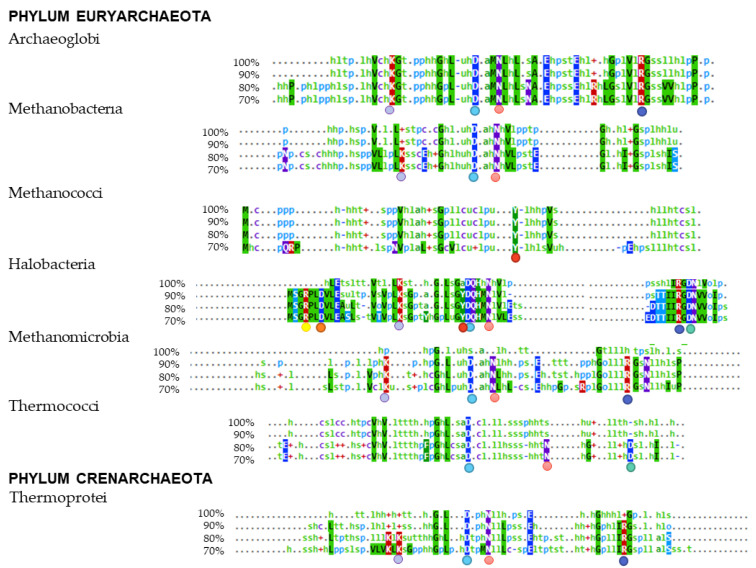
Consensus sequences of Lsm proteins according to taxonomic class by MView. Circles indicate RNA-binding residues. The external RNA binding site residues: Arg-4 (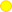), Asp-7 (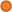), Tyr-34 (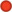). The internal RNA binding site residues: Lys-22 (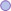), Asp-35 (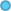), Asn-39 (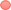), Arg-63 (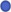), and Asp-65 (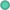).

Multiple alignments were performed according to the taxonomic class to which they belong, obtaining the consensus sequences shown in Figure 4. As shown, only 90% of the proteins of the Halobacteria class species have the conserved external RNA binding site residues Arg-4, Asp-7, and Tyr-34 located in the α-helix. As expected, residues Lys-22, Asp-35, and Asn-39 are conserved in most classes. In contrast, the residue Arg-63 is conserved in Archaeoglobi, Halobacteria, Methanomicrobia, and Thermoprotei, whereas the residue Asp-65 is conserved in species of the classes Halobacteria and Thermococci. The class Halobacteria has the most conserved RNA-binding residues identified in *P. abyssi* despite belonging to the different taxonomic classes. Apart from that, proteins belonging to the class Methanococci have only one conserved residue, which may indicate that they bind RNA differently to *P. abyssi*.

Moreover, as already mentioned, the Lsm protein of *H. gomorrense* (164 residues) is the only protein of the phylum Euryarchaeota with a size larger than 105 residues. After aligning this protein with both groups (smaller and larger Lsm proteins), it has been observed that it shows a high homology with the minor proteins from met-94. Therefore, it is most likely that the excessive length of this protein is due to an error in the assignment of the start codon.

### 3.5. Phylogenetic Analysis of the Lsm Proteins of the Archaea Domain

**Figure 5:**
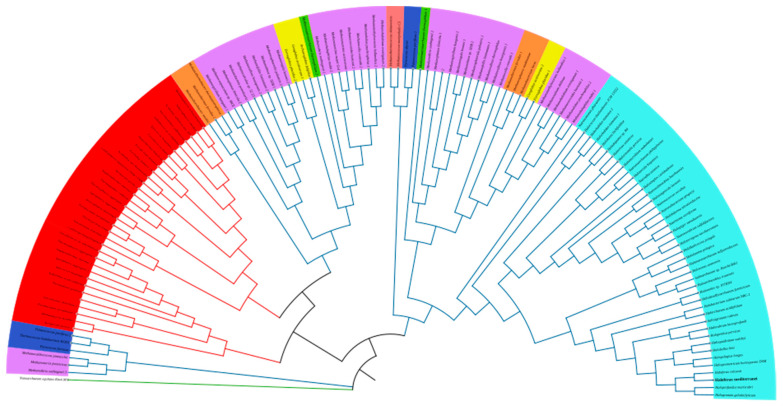
Phylogenetic tree based on 137 Lsm protein sequences (54–105 residues) belonging to species of the *Archaea* domain. Blue branches correspond to species of the Euryarchaeota phylum; green branch corresponds to the Nanoarchaeota phylum; and red branches correspond to the Crenarchaeota phylum. Species names are shaded according to the class to which they belong: Archaeoglobi (yellow), Methanobacteria (orange), Methanococci (pink), Methanomicrobia (purple), Methanonatroarchaeia (green), Thermocococci (dark blue), Halobacteria (light blue), and Thermoprotei (red).

To study how the Lsm proteins of the *Archaea* domain are distributed, 137 sequences were analyzed using the MEGA 11 software [32], which were aligned using Clustal W, and the phylogenetic tree was constructed using the Neighbor Joining statistical method and the Poisson model (Figure 5). As mentioned above, most of the class Halobacteria species have only one Lsm protein encoded in their genome (Figure 1A), which are clustered and closely related (Figure 5). 

On the other hand, the species of the class Methanomicrobia mostly have two Lsm proteins encoded in their genome (67% of the species) (Figure 1B). Despite belonging to the same class, the proteins are mainly separated into two groups: one more related to the Lsm proteins of the class Halobacteria and the other more related to the Lsm proteins of the phylum Crenarchaeota. Similar results have been observed for the Lsm proteins of species of the classes Methanonatroarchaeia and Archaeglobi. 

Most species of the class Methanobacteria have an Lsm protein encoded in their genome (80% of the species); however, these may be more closely related to Lsm proteins of the class Halobacteria or Lsm proteins of the phylum Crenarchaeota. In addition, Lsm proteins of the classes Methanomicrobia and Thermocci are evolutionarily distant from these two groups. Finally, the Lsm proteins of the phylum Crenarchaeota are grouped and closely related, forming two groups: one constituted exclusively by proteins of this phylum and the other related to proteins of the phylum Euryarchaeota, mainly of the order Methanomicrobia (Figure 5).

**Figure 6:**
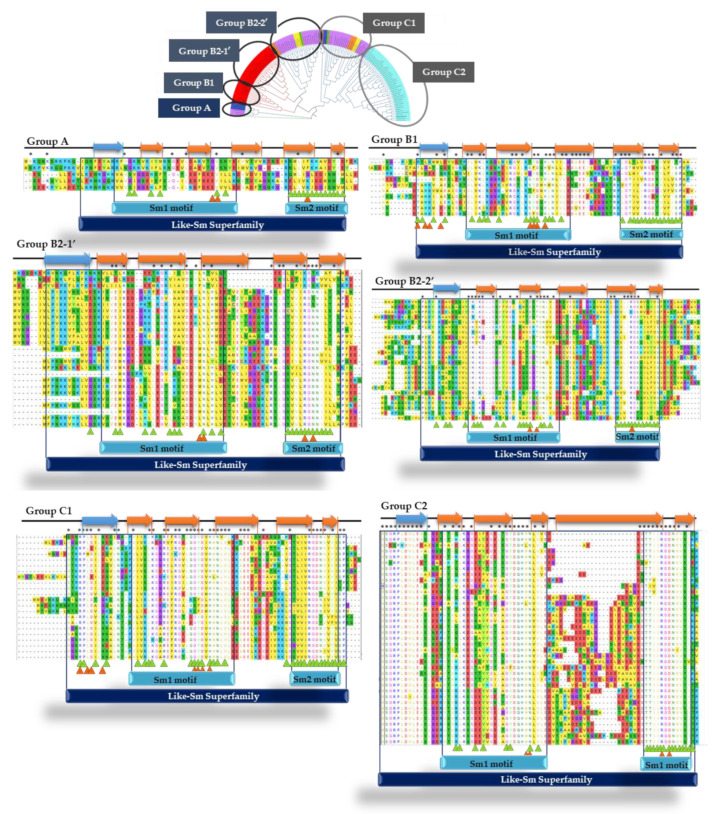
Analysis of the structural motifs of the Lsm protein groups from the phylogenetic tree. The conserved structural motifs are found inside the boxes. Conserved protein–protein interaction residues are indicated by green triangles, and protein–RNA interaction residues are indicated by orange triangles. Asterisks indicate conserved residues (>70%). Blue arrows correspond to the α-helix and orange arrows to the β-sheets. (*) Conserved residues.

A search for each group’s secondary structures and structural motifs was conducted to analyze the similarities and differences between the different groups of Lsm proteins obtained after constructing the phylogenetic tree (Figure 6). All Lsm proteins are characterized by two Sm motifs (Sm1 and Sm2), separated by a region varying in sequence and size, which are involved in protein–protein (indicated by green triangles) and protein–RNA (indicated by orange triangles) interactions. In all groups, Sm1 motif residues are primarily involved in protein–RNA interactions, while Sm2 motif residues are involved in protein–protein interactions. The Sm motif sequences are highly conserved within each group and subgroup and are very different between groups and subgroups. 

Moreover, the most remarkable sequence variability in composition and size is located in the β4 sheet. On the other hand, all of them are characterized by an α helix (represented by a blue arrow in Figure 6) followed by five β strands (represented by an orange arrow in Figure 6). In all groups, the Sm1 motif is formed by the β1 and β2 sheets, while the Sm2 motif is formed by part of the β4 sheet and the β5 sheet.

### 3.6. Analysis of Protein–Protein Interaction Networks of Lsm Proteins

**Figure 7:**
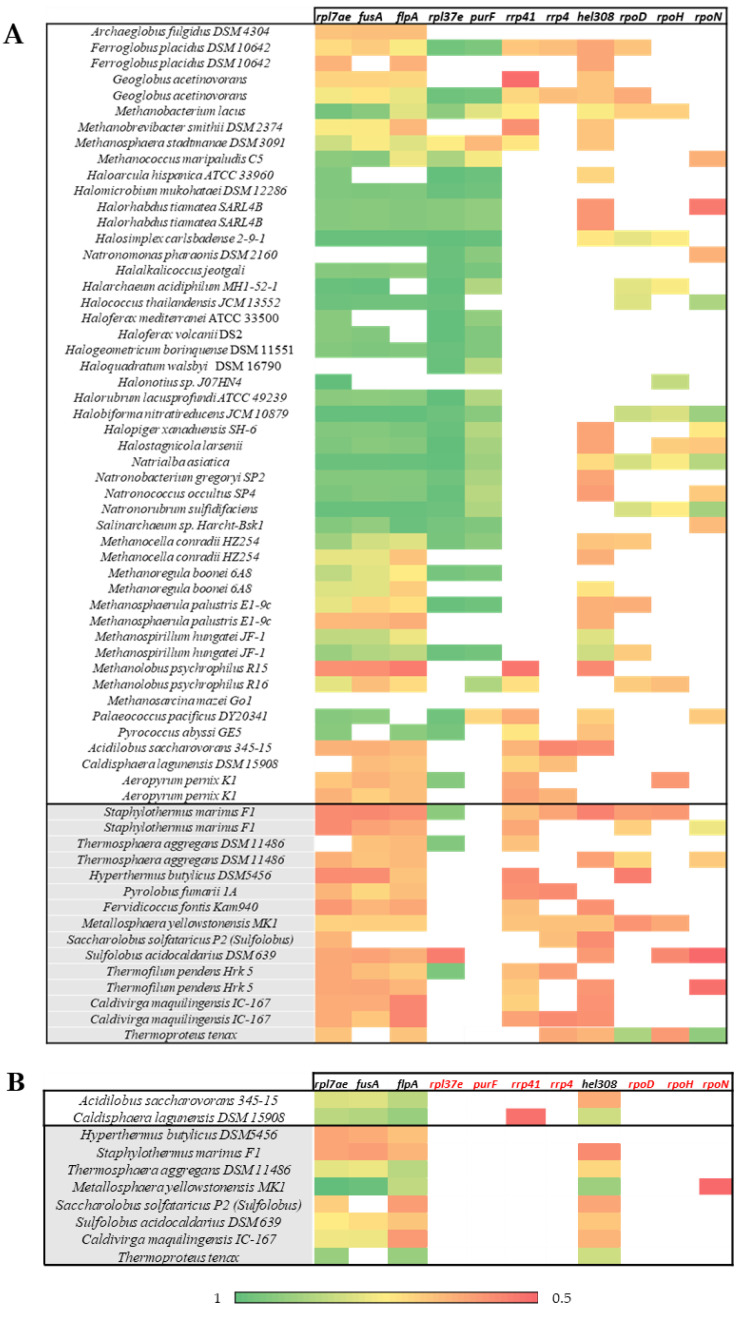
Heat map from data deposited in STRING. (**A**) Size ≤ 105 residues. (**B**) Size ≥ 139 residues. The colors indicate the probability of interaction: very high probability (green), high probability (yellow), and medium probability (red). Species of the phylum Euryarchaeota (white) and Crenarchaeota (grey).

The STRING 11.0 bioinformatics tool [25] predicts gene–gene or protein–protein associations derived from experimental and bibliographic information, i.e., the interactome. For this purpose, all Lsm proteins in Table S1 were searched, of which 74 were deposited in this database. Figure 7 shows 11 genes that appear in the vast majority of the species analyzed: *rpl7ae* and *rpl37e*, which encode 50S ribosomal proteins; *fusA*, which encodes elongation factor 2; *flpA*, which encodes fibrillin-like pre-rRNA processing protein; *purF*, which encodes amidophosphoribosyltransferase; *rrp4* and *rrp41,* which encode RNA-binding proteins of the exosome complex; *hel308*, which encodes a helicase; and *rpoD, rpoH,* and *rpoN*, which encode different RNA polymerase subunits (Figure 7A). All these genes encode proteins closely related to the RNA metabolism.

The ribosomal protein L7Ae is a multifunctional RNA-binding protein that recognizes the K-turn motif of the ribosome and the H/ACA and C/D boxes of sRNAs, generating conformational changes in sRNAs [60]. As mentioned above, the ribosomal protein L37e is found adjacent to the *lsm* gene in many archaea species. Its primary function is stabilizing interactions between the domains to maintain the structural integrity of the 50S subunit through interactions with RNA [54]. The *fusA* gene encodes translational elongation factor 2, which has homologs in all three domains of life, EF-G in bacteria, eEF-2 in eukaryotes, and aEF-2 in archaea; it is composed of five domains, a GTPase domain, and domains II to V; and mediates the hydrolysis of a GTP molecule during translocation [61,62]. 

The fibrillin-like pre-mRNA processing protein (*flpA* gene) is involved in pre-rRNA and tRNA processing, using the methyl group of S-adenosyl-L-methionine to catalyze 2’-hydroxyl methylation on rRNA and tRNA [63]. The proteins Rrp4 and Rrp41 are part of the exosome, which plays an essential role in RNA processing and degradation. In archaea and eukaryotes, the exosome structure consists of a hexameric core composed of three Rrp41–Rrp42 dimers and a trimeric cap formed by Csl4 or Rrp4 proteins. The archaeal exosome is catalytically active, localizing to the Rrp41 subunits inside the hexameric core, while the Rrp4 trimer increases the catalytic efficiency of the enzyme complex [64,65]. 

The *hel308* gene encodes the SF2 helicase (superfamily 2), with homologs in eukaryotes but not in bacteria, and exhibits DNA-dependent ATPase and helicase activity, involved in replication fork repair. It has high sequence similarity to Ski2 helicases involved in releasing RNA molecules for degradation [66]. Finally, the *rpoD, rpoH,* and *rpoN* genes encode distinct RNA polymerase subunits, catalyzing DNA transcription into RNA [67]. All these proteins are closely related to the RNA metabolism [54,61,62,63,64,65,66,67]. That reinforces the hypothesis that Lsm proteins are closely associated to the RNA metabolism.

Interestingly, there is no evidence that the larger Lsm proteins (Figure 7B) can interact with the ribosomal protein L37e, proteins that are part of the exosome enzyme complex, or with the different RNA polymerase subunits. It is likely that, despite being annotated as Lsm proteins, they perform different functions and/or act through other mechanisms of action.

## 4. Conclusions

The different bioinformatic tools employed in this work have increased the knowledge of Lsm proteins in the *Archaea* domain. All archaeal species analyzed encode one to three Lsm proteins in their genomes. Most species of the phylum Euryarchaeota present only one Lsm protein, while most species of the phylum Crenarchaeota present two Lsm proteins. The Lsm proteins of the phylum Crenarchaeota are classified into two groups based on molecular weight. Many of these genes are adjacent to transcriptional regulators of the Lrp/AsnC and MarR families, RNA-binding proteins, and ribosomal protein L37e. 

Notably, only proteins from species of the class Halobacteria conserved the internal and external residues of the RNA-binding site identified in *P. abyssi*. Finally, in most species, the *lsm* genes show associations with genes that encode proteins closely related to the RNA metabolism. The role of Lsm in *Archaea* remains unknown; however, they appear to play a key role in the RNA metabolism. More work is needed to elucidate the action mechanism of Lsm proteins, such as the construction of deletion mutants and their characterization, studies of the regulation of small RNAs, e.g., by crosslinking and sequencing the putative small RNAs (RIL-seq), or of proteins, e.g., by copurification.

## Data Availability

Data is contained within the article and Supplementary Materials.

## Supplementary Materials

The following supporting information can be downloaded at: https://www.mdpi.com/article/10.3390/microorganisms11051196/s1. Table S1: List of selected Lsm proteins from the *Archaea* domain; Table S2: Parameters obtained by ProtParam (Expasy) of the 163 proteins analyzed from the *Archaea* domain; Table S3: Gene environment of the lsm gene in the different genomes of species of the *Archaea* domain; Table S4: Overlap analysis of *lsm* and *rpl37e* genes in 80 species of the phylum Euryarchaeota.
