# Supplementary material for: Comprehensive Bioinformatics Analysis of the Biodiversity of Lsm Proteins in the Archaea Domain
Source: Microorganisms. 2023 May 3;11(5):1196. doi: 10.3390/microorganisms11051196 (PMC10221803; doi:10.3390/microorganisms11051196)
Supplement: Supplementary file 1 [file microorganisms-11-01196-s001.zip › microorganisms-2290906-supplementary.pdf]

Supplementary Materials

Table S1. List of selected Lsm proteins from the *Archaea* domain. Code: corresponds to the serial number assigned in this work. The species with multiple encoded proteins being indicated by ".1", ".2" or ".3"

Code	Species name	Description	Uniprot	Gene name
**Phylum Nanoarchaeota – Nanoarchaeales - Nanoarchaeaceae**				
**1**	*Nanoarchaeum equitans* Kin4-M	Putative U6 small nuclear ribonucleoprotein	Q74N54	NEQ037
**Phylum Euryarchaeota**				
**Archaeoglobi – Archaeoglobales - Archaeoglobaceae**				
**2**	*Archaeglobus fulgidus* DSM 4304	Like-Sm ribonucleoprotein core	A0A075WCA7	AFULGI_0000 3630
**3.1**	*Ferroglobus placidus* DSM 10642	Like-Sm ribonucleoprotein core	D3RZS2	Ferp_1842
**3.2**	*Ferroglobus placidus* DSM 10642	Like-Sm ribonucleoprotein core	D3S2K3	Ferp_0354
**4.1**	*Geoglobus acetinovorans*	Like-Sm ribonucleoprotein core	A0A0A7GIA4	GACE_1661
**4.2**	*Geoglobus acetinovorans*	Small nuclear ribonucleoprotein	A0A0A7GE96	GACE_1349
**Methanobacteria - Methanobacteriales - Methanobacteriaceae**				
**5**	*Methanobacterium lacus*	snRNP Sm-like protein	F0TBX8	Metbo_2101
**6**	*Methanobrevibacter smithii DSM 2374*	LSM domain protein	D2ZP11	METSMIF1_02 568
**7**	*Methanosphaera stadtmanae DSM 3091*	Putative snRNP Sm-like protein	Q2NF47	Msp_1175
**8**	*Methanothermobacter thermautotrophicus* Delta H	Unknown	O27489	MTH_1440
**Methanobacteria - Methanobacteriales -Methanothermaceae**				
**9.1**	*Methanothermus fervidus* DSM 2088	Small nuclear ribonucleoprotein, LSM family	E3GXZ5	Mfer_0375
**9.2**	*Methanothermus fervidus* DSM 2088	Like-Sm ribonucleoprotein core	E3GWX1	Mfer_0056
**Methanococci – Methanococcales - Methanocaldococcaceae**				
**10**	*Methanocaldococcus jannaschii*	RNA chaperone Hfq	Q58830	MJ1435
**11**	*Methanotorris formicicus*	Hypothetical protein	H1KZ28	MetfoDRAFT_ 1051
**Methanococci – Methanococcales - Methanococcaceae**				
**12**	*Methanococcus maripaludis* C5	Small nuclear ribonucleoprotein, LSM family	A4FX23	MmarC5_0437
**13**	*Methanothermococcus okinawensis*	Like-Sm ribonucleoprotein core	F8ANN5	Metok_0240
**Methanonatronarchaeia - Methanonatronarchaeales - Methanonatronarchaeaceae**				
**14.1**	*Methanonatronarchaeum thermophilum*	Small nuclear ribonucleoprotein	A0A1Y3GBK1	AMET1_0450
**14.2**	*Methanonatronarchaeum thermophilum*	Small nuclear ribonucleoprotein	A0A1Y3GJ83	AMET1_0176
**Methanopyri – Methanopyrales - Methanopyraceae**				
**15**	*Methanopyrus kandleri* AV19	Small nuclear ribonucleoprotein (snRNP) homolog	Q8TYS2	lsm1 MK0220
**Halobacteria – Halobacteriales - Haloarculaceae**				
**16**	*Haloarcula hispanica* ATCC 33960	Small nuclear ribonucleoprotein	G0HR69	snp HAH_1444
	Halomicroarcula	There are no sequenced genomes of species in this genus.		
**17**	*Halomicrobium mukohataei* DSM 12286	Like-Sm ribonucleoprotein core	C7NWK0	Hmuk_2097
**18.1**	*Halorhabdus tiamatea* SARL4B	snRNP-like protein	S6CZE0	HTIA_0589

**18.2**	*Halorhabdus tiamatea* SARL4B	snRNP-like protein	U2DN39	snp HLRTI_00 0706
**19**	*Halorientalis persicus*	Small nuclear ribonucleoprotein, LSM family	A0A1H8JVI5	SAMN0521638 8_1005184
	Halosiccatus	There are no sequenced genomes of species in this genus.		
**20**	*Halosimplex carlsbadense* 2-9-1	Sm ribonucleoprotein-like protein	M0CQM3	C475_11920
**21**	*Natronomonas pharaonis* DSM 2160	RNA-binding protein Lsm	A0A1U7EYC9	lsm NP_4308A
**Halobacteria – Halobacteriales - Halobacteriaceae**				
**22**	*Haladaptatus sp.* R4	Like-Sm ribonucleoprotein core	A0A166RF41	A4G99_17290
**23**	*Halalkalicoccus jeotgali*	Small nuclear ribonucleoprotein	D8J3B1	HacjB3_09175, C497_13503
**24**	*Halanaeroarchaeum sulfurireducens*	Like-Sm ribonucleoprotein core	A0A0F7P9T0	HLASF_1417
**25**	*Halarchaeum acidiphilum* MH1-52-1	Small nuclear ribonucleoprotein	U2YR27	MBEHAL_018 5
**26**	*Haloarchaeobius iranensis*	Small nuclear ribonucleoprotein, LSM family	A0A1G9Z5M9	SAMN0519255 4_11837
**27**	*Halobacterium salinarum* NRC-1	Small nuclear ribonucleoprotein	Q9HPS2	VNG_1496G
	Halocalculus	There are no sequenced genomes of species in this genus.		
**28**	*Halodesulfurarchaeum formicicum*	Like-Sm ribonucleoprotein core	A0A1D8S3F8	HSR6_0723, HTSR_0697
	Halomarina oriensis	There are no Lsm proteins encoded in the genome.		
	Halorubellus	There are no sequenced genomes of species in this genus.		
	Halorussus rarus	There are no Lsm proteins encoded in the genome.		
	Halostella salina	There are no Lsm proteins encoded in the genome.		
**29**	*Halovenus aranensis*	Small nuclear ribonucleoprotein, LSM family	A0A1G8XHR6	SAMN0521622 6_11185
**30**	*Natronoarchaeum philippinense*	Small nuclear ribonucleoprotein, LSM family	A0A285N388	SAMN0626918 5_0401
	Salarchaeum	There are no sequenced genomes of species in this genus.		
	Salinirubrum	There are no sequenced genomes of species in this genus.		
	Salinirussus	There are no sequenced genomes of species in this genus.		
**Halobacteria – Halobacteriales - Halococcaceae**				
**31**	*Halococcus thailandensis* JCM 13552	Like-Sm ribonucleoprotein core	M0N3E1	C451_15240
**Halobacteria –Haloferacales - Haloferacaceae**				
**32**	*Halobellus limi*	Small nuclear ribonucleoprotein, LSM family	A0A1H5U874	SAMN0448813 3_0510
**33**	*Haloferax mediterranei* ATCC 33500	Like-Sm ribonucleoprotein core	I3R850	lsm1 HFX_2733
**34**	*Haloferax volcanii* DS2	RNA-binding protein Lsm	D4GW47	lsm HVO_2723
**35**	*Halogeometricum borinquense* DSM 11551	Small nuclear ribonucleoprotein, LSM family	E4NR43	Hbor_11900, C499_03438
**36**	*Halogranum gelatinilyticum*	Small ribonucleoprotein	A0A1G9SXM6	SAMN0448794 9_1540
**37**	*Halopelagius longus*	Like-Sm ribonucleoprotein core	A0A1H0Z2L0	DWB78_14205, SAMN0521627 8_0962
	Haloplanus aerogenes	There are no Lsm proteins encoded in the genome.		
**38**	*Haloprofundus marisrubri*	Like-Sm ribonucleoprotein core	A0A0W1R5K3	AUR64_18700
**39**	*Haloquadratum walsbyi* DSM 16790	RNA-binding protein Lsm	Q18GA2	HQ_2891A
**Halobacteria – Haloferacales - Halorubraceae**				

**40**	*Halobaculum gomorrense*	Small ribonucleoprotein (SnRNP) homolog	A0A1M5SJ24	SAMN05443636_2486
	Halobium	There are no sequenced genomes of species in this genus.		
**41**	*Halohasta litchfieldiae*	Small nuclear ribonucleoprotein, LSM family	A0A1H6REP0	halTADL_2981
**42**	*Halolamina pelagica*	Like-Sm ribonucleoprotein core	A0A0P7GS73	SY89_02478
**43**	*Halonotius sp.* J07HN4	Small nuclear ribonucleoprotein, LSM family	U1QFN8	J07HN4v3_01221
	Haloparvum sedimenti	There are no Lsm proteins encoded in the genome.		
**44**	*Halopenitus persicus*	Small nuclear ribonucleoprotein, LSM family	A0A1H3DLY1	SAMN05216564_10161
**45**	*Halorubrum lacusprofundi* ATCC 49239	Like-Sm ribonucleoprotein core	B9LQK3	Hlac_2046
**Halobacteria – Natrialbales - Natrialbaceae**				
**46**	*Salinigranum rubrum*	Like-Sm ribonucleoprotein core	A0A2I8VK88	C2R22_12240
**47**	*Halobiforma nitratireducens* JCM 10879	Like-Sm ribonucleoprotein core	M0LWP4	C446_11317
**48**	*Halopiger xanaduensis* SH-6	Like-Sm ribonucleoprotein core	F8DBH4	Halxa_2470
**49**	*Halostagnicola larsenii*	Like-Sm ribonucleoprotein core	W0JQW5	HALLA_12960
**50**	*Haloterrigena saccharevitans*	Like-Sm ribonucleoprotein core	A0A1S8ATE8	A6E15_03575
	Halovarius	There are no sequenced genomes of species in this genus.		
**51**	*Halovivax asiaticus* JCM 14624	Like-Sm ribonucleoprotein core	M0BA34	C479_15025
	Natrarchaeobius chitinivorans	There are no Lsm proteins encoded in the genome.		
**52**	*Natrialba asiatica*	Like-Sm ribonucleoprotein core	M0AV87	C481_07291
	Natribaculum	There are no sequenced genomes of species in this genus.		
**53**	*Natrinema versiforme*	Like-Sm ribonucleoprotein core	L9Y0R1	C489_09792
**54**	*Natronobacterium gregoryi* SP2	Small nuclear ribonucleoprotein	L0AJN2	Natgr_2077, C490_05102, CYV19_03665
	Natronobiforma	There are no sequenced genomes of species in this genus.		
**55**	*Natronococcus occultus* SP4	Small nuclear ribonucleoprotein	L0JZL9	Natoc_2733
**56**	*Natronolimnobius baerhuensis*	Like-Sm ribonucleoprotein core	A0A202EBV8	B2G88_02805
**57**	*Natronorubrum sulfidifaciens*	Like-Sm ribonucleoprotein core	L9WCP1	C495_03237
**58**	*Salinarchaeum sp.* Harcht-Bsk1	Small nuclear ribonucleoprotein	R4W1T9	L593_13435
	Saliphagus sp. LR7 16-66	There are no sequenced genomes of species in this genus.		
**Methanomicrobia – Methanocellales – Methanocellaceae**				
**59.1**	*Methanocella conradii* HZ254	Small nuclear ribonucleoprotein, LSM family	H8I4X9	Mtc_2340
**59.2**	*Methanocella conradii* HZ254	Small nuclear ribonucleoprotein, LSM family	H8I9S9	Mtc_1786
**Methanomicrobia - Methanomicrobiales - Methanocalculaceae**				
**60**	*Methanocalculus sp. 52_23*	Like-Sm ribonucleoprotein, core	A0A101H6G2	XD88_0189
**Methanomicrobia - Methanomicrobiales – Methanocorpusculaceae**				
**61**	*Methanocorpusculum sp.* MCE	Putative snRNP Sm-like protein	A0A366MEZ9	ALMCE001_14400
**Methanomicrobia - Methanomicrobiales - Methanomicrobiaceae**				
**62**	*Methanoculleus thermophilus*	RNA-binding protein	A0A1G9A972	SAMN04488571_105233
**63.1**	*Methanofollis liminatans* DSM 4140	Like-Sm ribonucleoprotein core	J1L538	Metli_2309
**63.2**	*Methanofollis liminatans* DSM 4140	Like-Sm ribonucleoprotein core	J0S8E7	Metli_0886
**64.1**	*Methanogenium bourgense*	Sm ribonucleoprotein	I7LL38	BN140_0111
**64.2**	*Methanogenium bourgense*	Archaeal Like-Sm protein	I7LMG4	BN140_1301
**65.1**	*Methanolacinia petrolearia* DSM	Like-Sm ribonucleoprotein core	E1RG85	Mpet_2656

	11571			
**65.2**	*Methanolacinia petrolearia* DSM 11571	Like-Sm ribonucleoprotein core	E1RHD7	Mpet_1688
	Methanomicrobium mobile	There are no Lsm proteins encoded in the genome.		
**66.1**	*Methanoplanus limicola* DSM 2279	Small nuclear ribonucleoprotein, LSM family	H1Z321	Metlim_1458
**66.2**	*Methanoplanus limicola* DSM 2279	Small nuclear ribonucleoprotein, LSM family	H1YXV4	Metlim_1852
**Methanomicrobia - Methanomicrobiales - Methanoregulaceae**				
**67.1**	*Methanolinea sp.* SDB	Ribonucleoprotein	A0A0Q0VVX7	APR55_05850
**67.2**	*Methanolinea sp.* SDB	RNA-binding protein	A0A0Q0VTJ3	APR55_00935
**68.1**	*Methanoregula boonei* 6A8	Like-Sm ribonucleoprotein core	A7I4F6	Mboo_0093
**68.2**	*Methanoregula boonei* 6A8	Like-Sm ribonucleoprotein core	A7I8R9	Mboo_1613
**69.2**	*Methanosphaerula palustris* E1-9c	Like-Sm ribonucleoprotein core	B8GJW6	Mpal_0396
**69.2**	*Methanosphaerula palustris* E1-9c	Like-Sm ribonucleoprotein core	B8GIP1	Mpal_1541
**Methanomicrobia - Methanomicrobiales - Methanospirillaceae**				
**70.1**	*Methanospirillum hungatei* JF-1	Small nuclear ribonucleoprotein, LSM family	Q2FM21	Mhun_1205
**Methanomicrobia – Methanosarcinales - Methanosaetaceae**				
**71.1**	*Methanothrix soehngenii* GP6	LSM domain protein	F4BUV3	MCON_1727
**71.2**	*Methanothrix soehngenii* GP6	LSM domain protein	F4C031	MCON_1204
**71.3**	*Methanothrix soehngenii* GP6	RNA chaperone Hfq	F4BVY6	MCON_0386
**Methanomicrobia – Methanosarcinales - Methanosarcinaceae**				
	Methanimicrococcus	There are no sequenced genomes of species in this genus.		
**72.1**	*Methanococcoides burtonii*	Like-Sm ribonucleoprotein	Q12U30	Mbur_2181
**72.2**	*Methanococcoides burtonii*	LSM domain-containing protein	Q12ZD8	Mbur_0177
**73.1**	*Methanohalobium evestigatum* Z-7303	Like-Sm ribonucleoprotein core	D7E692	Metev_0183
**73.2**	*Methanohalobium evestigatum* Z-7303	Like-Sm ribonucleoprotein core	D7EA61	Metev_1901
**73.3**	*Methanohalobium evestigatum* Z-7303	Hypothetical protein Metev_0717	D7E6Z4	Metev_0717
**74.1**	*Methanohalophilus mahii* DSM 5219	Small nuclear ribonucleoprotein, LSM family	D5E9P3	Mmah_0362
**74.2**	*Methanohalophilus mahii* DSM 5219	Small nuclear ribonucleoprotein, LSM family	D5EAU8	Mmah_0775
**75.1**	*Methanolobus psychrophilus* R15	Small ribonucleoprotein	K4M888	Mpsy_0252
**75.2**	*Methanolobus psychrophilus* R15	Putative snRNP Sm-like protein	K4MIF5	Mpsy_2781
**76.1**	*Methanomethylovorans hollandica* DSM 15978	Small nuclear ribonucleoprotein	L0KY05	Metho_0713
**76.2**	*Methanomethylovorans hollandica* DSM 15978	Small nuclear ribonucleoprotein	L0L0H3	Metho_1555
**77.1**	*Methanosalsum zhilinae* DSM 4017	Like-Sm ribonucleoprotein core	F7XNG0	Mzhil_1368
**77.2**	*Methanosalsum zhilinae* DSM 4017	Like-Sm ribonucleoprotein core	F7XKW7	Mzhil_0803
**78**	*Methanosarcina acetivorans* C2A	Sm protein	Q8TQX4	MA_1413
**79**	*Methanosarcina mazei* Go1	Small nuclear riboprotein-like protein	Q8PUF1	MM_2383
**Methanomicrobia - Methanosarcinales - Methermicoccaceae**				
	Methermicoccus shengliensis	There are no Lsm proteins encoded in the genome.		
**Thermococci –Thermococcales - Thermococcaceae**				
**80.1**	*Palaeococcus pacificus* DY20341	Putative snRNP Sm-like protein	A0A075LSS7	PAP_02985

**80.2**	*Palaeococcus pacificus* DY20341	Uncharacterized protein	A0A075LSE3	PAP_06315
**81**	*Pyrococcus abyssi* GE5	small nuclear ribonucleoprotein	Q9V0Y8	PYRAB06500, PAB8160
**82**	*Pyrococcus furiosus*	hypothetical protein	I6URS7	PFC_08805
**83**	*Thermococcus kodakarensis* KOD1	hypothetical protein	Q5JI86	TK0933
**Phylum Crenarchaeota**				
**Thermoprotei - Acidilobales - Acidilobaceae**				
**84.1**	*Acidilobus saccharovorans* 345-15	Putative small nucleolar RNP protein Sm	D9Q0Y5	ASAC_0566
**84.2**	*Acidilobus saccharovorans* 345-15	Like-Sm ribonucleoprotein, core	D9Q078	ASAC_0309
**Thermoprotei- Acidilobales - Caldisphaeraceae**				
**85.1**	*Caldisphaera lagunensis* DSM 15908	small nuclear ribonucleoprotein	L0AA67	Calag_1034
**85.2**	*Caldisphaera lagunensis* DSM 15908	small nuclear ribonucleoprotein	L0AD38	Calag_1248
**Thermoprotei- Desulfurococcales - Desulfurococcaceae**				
**86.1**	*Aeropyrum pernix* K1	Small nucleolar RNP protein Sm	Q9YEQ5	APE_0525a, APES022
**86.2**	*Aeropyrum pernix* K1	Putative small nucleolar RNP protein Sm	Q05E36	APE_0957a
**87.1**	*Desulfurococcus amylolyticus* DSM 16532	Like-Sm ribonucleoprotein core	I3XTI4	Desfe_1393
**87.2**	*Desulfurococcus amylolyticus* DSM 16532	Small nuclear ribonucleoprotein, LSM family	I3XQ67	Desfe_0180
**87.3**	*Desulfurococcus amylolyticus* DSM 16532	Like-Sm ribonucleoprotein core	I3XTF2	Desfe_1360
**88.1**	*Ignicoccus hospitalis* KIN4/1	Like-Sm ribonucleoprotein core	A8AB56	Igni_0978
**88.2**	*Ignicoccus hospitalis* KIN4/1	Like-Sm ribonucleoprotein core	A8A8T9	Igni_0157
**89.1**	*Ignisphaera aggregans* DSM 17230	Like-Sm ribonucleoprotein core	E0SPE5	Igag_0035
**89.2**	*Ignisphaera aggregans* DSM 17230	Like-Sm ribonucleoprotein core	E0SQZ9	Igag_1527
**89.3**	*Ignisphaera aggregans* DSM 17230	Like-Sm ribonucleoprotein core	E0SPS7	Igag_0097
**90.1**	*Staphylothermus marinus* F1	Small nuclear ribonucleoprotein, LSM family	A3DN32	Smar_0943
**90.2**	*Staphylothermus marinus* F1	Small nuclear ribonucleoprotein, LSM family	A3DP35	Smar_1304
**90.3**	*Staphylothermus marinus* F1	Like-Sm ribonucleoprotein core	A3DMZ9	Smar_0910
	Stetteria	There are no sequenced genomes of species in this genus.		
	Sulfophobococcus	There are no sequenced genomes of species in this genus.		
	Thermodiscus	There are no sequenced genomes of species in this genus.		
**91.1**	*Thermogladius calderae* 1633	LSM family small nuclear ribonucleoprotein	I3TCF2	TCELL_0015
**91.2**	*Thermogladius calderae* 1633	Like-Sm ribonucleoprotein core	I3TD69	TCELL_0282
**92.1**	*Thermosphaera aggregans* DSM 11486	Small nuclear ribonucleoprotein, LSM family	D5U314	Tagg_1248
**92.2**	*Thermosphaera aggregans* DSM 11486	Small nuclear ribonucleoprotein, LSM family	D5U030	Tagg_0200
**92.3**	*Thermosphaera aggregans* DSM 11486	Like-Sm ribonucleoprotein core	D5U347	Tagg_1284
**93.1**	*Zestosphaera tikiterensis*	Sm ribonucleo	A0A2R7Y5M3	B7O98_05210
**93.2**	*Zestosphaera tikiterensis*	Hypothetical protein	A0A2R7Y6G7	B7O98_01430

Thermoprotei - Desulfurococcales – Pyrodictiaceae				
	Geogemma	There are no sequenced genomes of species in this genus.		
**94.1**	*Hyperthermus butylicus* DSM5456	Putative snRNP Sm-like protein	A2BIZ9	Hbut_0084
**94.2**	*Hyperthermus butylicus* DSM5456	Hypothetical protein	A2BK21	Hbut_0468
**95.1**	*Pyrodictium delaneyi*	Small nuclear ribonucleoprotein, LSM family	A0A0P0N236	Pyrde_0619
**95.2**	*Pyrodictium delaneyi*	Hypothetical protein	A0A0P0N1L9	Pyrde_0065
**96**	*Pyrolobus fumarii* 1A	Like-Sm ribonucleoprotein core	G0EEI3	Pyrfu_0152
**Thermoprotei – Fervidicoccales - Fervidicoccaceae**				
**97**	*Fervidicoccus fontis* Kam940	Like-Sm ribonucleoprotein core	H9ZZX2	FFONT_0289
**Thermoprotei - Sulfolobales – Sulfolobaceae**				
**98.1**	*Acidianus sulfidivorans* JP7	Sm ribonucleo	A0A2U9IMU8	DFR86_07025
**98.2**	*Acidianus sulfidivorans* JP7	Sm ribonucleo	A0A2U9IMK4	DFR86_06530
**99.1**	*Metallosphaera yellowstonensis* MK1	Small nuclear ribonucleoprotein	H2C6B2	MetMK1DRAFT_00020890
**99.2**	*Metallosphaera yellowstonensis* MK1	Small nuclear ribonucleoprotein	H2C6Q1	MetMK1DRAFT_00022410
**100.1**	*Sulfolobus* ***solfataricus*** **P2**	Small nuclear riboprotein protein (snRNP-1)	Q980S8	snRNP-1SSO5410
**100.2**	*Sulfolobus* ***solfataricus*** **P2**	Hypothetical protein	Q980L6	SSO0276
	Stygiolobus	There are no sequenced genomes of species in this genus.		
**101.1**	*Sulfodiicoccus acidiphilus*	Sm ribonucleoprotein	A0A348B2S4	HS1genome_0865
**101.2**	*Sulfodiicoccus acidiphilus*	Sm ribonucleoprotein	A0A348B1C8	HS1genome_0369
**102.1**	*Sulfolobus acidocaldarius* DSM 639	Hypothetical protein	Q4J9G0	Saci_1224
**102.2**	*Sulfolobus acidocaldarius* DSM 639	Conserved Archaeal protein	Q4JAY5	Saci_0660
**103.1**	*Sulfurisphaera tokodaii str.* 7	Archaeal Sm protein	F9VMQ9	sm2 STK_02355, STS030
**103.2**	*Sulfurisphaera tokodaii str.* 7	Archaeal Sm protein	F9VMU2	sm3 ST0326, STK_03260
**Thermoprotei - Thermoproteales - Thermofilaceae**				
**104.1**	*Thermofilum pendens* Hrk 5	Like-Sm ribonucleoprotein, core	A1RXA0	Tpen_0421
**104.2**	*Thermofilum pendens* Hrk 5	Like-Sm ribonucleoprotein core	A1RY58	Tpen_0736
**Thermoprotei - Thermoproteales - Thermoproteaceae**				
**105.1**	*Caldivirga maquilingensis* IC-167	Like-Sm ribonucleoprotein core	A8M9D3	Cmaq_1529
**105.2**	*Caldivirga maquilingensis* IC-167	Like-Sm ribonucleoprotein core	A8MD45	Cmaq_0866
**105.3**	*Caldivirga maquilingensis* IC-167	Like-Sm ribonucleoprotein core	A8M9J8	Cmaq_0025
**106.1**	*Pyrobaculum aerophilum* DSM 7523	Small nuclear ribonucleoprotein homolog (Sm-like)	Q8ZYG5	PAE0790
**106.2**	*Pyrobaculum aerophilum* DSM 7523	Small nuclear ribonucleoprotein homolog (Sm-like)	Q8ZYP8	PAE0676
**106.3**	*Pyrobaculum aerophilum* DSM 7523	Small nuclear ribonucleoprotein homolog (Sm-like)	Q8ZVU2	PAE2122
**107**	*Thermocladium sp.* ECH_B	Small nuclear ribonucleoprotein (Sm)	A0A117SUX3	AT710_06915
**108.1**	*Thermoproteus tenax*	Small nuclear ribonucleoprotein (Sm)	G4RL43	TTX_1667
**108.2**	*Thermoproteus tenax*	Small nuclear ribonucleoprotein	G4RLV2	TTX_1933

		(Sm)		
**109.1**	*Vulcanisaeta distribute* DSM 14429	Like-Sm ribonucleoprotein core	E1QRI0	Vdis_2428
**109.2**	*Vulcanisaeta distribute* DSM 14429	Sm ribonucleoprotein core-like protein	E1QSA2	Vdis_0080
**109.3**	*Vulcanisaeta distribute* DSM 14429	Like-Sm ribonucleoprotein core	E1QV44	Vdis_1863

Table S2. Parameters obtained by ProtParam (Expasy) of the 163 proteins analysed from the *Archaea* domain. Number of amino acids (Aa); molecular weight (MW); pI; number of negatively charged residues (Asp + Glu); number of positively charged residues (Arg + Lys); extinction coefficient; instability index; stability; aliphatic index; grand average hydropathy value (GRAVY).

Code	Species name	Aa	MW (Da)	pI	Asp + Glu	Arg + Lys	Extinction coefficient (M^{-1} cm^{-1})	Instability index	Stability	Aliphatic index	GRAVY
**1**	*Nanoarchaeum equitans Kin4-M*	66	7621,08	8,01	10	11	2980	29,57	stable	118,03	-0,018
**2**	*Archaeglobus fulgidus DSM 4304*	74	8380,79	4,88	12	9	2980	33,2	stable	99,86	-0,309
**3.1**	*Ferroglobus placidus DSM 10642*	73	8031,37	8,16	9	10	1490	28,58	stable	115,89	0,064
**3.2**	*Ferroglobus placidus DSM 10642*	74	8451,73	4,86	13	9	4470	33,1	stable	106,49	-0,293
**4.1**	*Geoglobus acetinovorans*	65	7509,69	5	11	8	2980	21,01	stable	98,77	-0,34
**4.2**	*Geoglobus acetinovorans*	73	8065,41	6,55	9	9	1490	36,56	stable	118,63	0,125
**5**	*Methanobacterium lacus*	80	8922,42	6,56	10	10	1490	41,52	unstable	120,5	-0,02
**6**	*Methanobrevibacter smithii DSM 2374*	65	7264,19	4,69	11	8	1490	16,85	stable	109,23	-0,398
**7**	*Methanosphaera stadtmanae DSM 3091*	81	8885,12	5,67	10	9	1490	41,89	unstable	98,64	-0,332
**8**	*Methanothermobacter thermautotrophicus Delta H*	67	7780,08	9,57	8	12	1490	23,09	stable	91,64	-0,479
**9.1**	*Methanothermus fervidus DSM 2088*	80	9119,62	8,09	11	12	1490	39,89	stable	110,75	-0,323
**9.2**	*Methanothermus fervidus DSM 2088*	65	7655,95	5,05	12	10	2980	17,72	stable	97,38	-0,286
**10**	*Methanocaldococcus jannaschii*	71	8294,76	9,74	8	14	5960	27,26	stable	95,92	-0,456
**11**	*Methanotorris formicicus*	76	9013,56	9,7	9	15	5960	27,59	stable	83,16	-0,537
**12**	*Methanococcus maripaludis C5*	72	8136,45	5,71	11	10	2980	10,27	stable	101,39	-0,232

Code	Species name	Aa	MW (Da)	pI	Asp + Glu	Arg + Lys	Extinction coefficient (M^{-1} cm^{-1})	Instability index	Stability	Aliphatic index	GRAVY
**13**	*Methanothermococcus okinawensis*	71	7878,23	6,04	10	9	1920	13,79	stable	122,11	-0,077
**14.1**	*Methanonatronarchaeum thermophilum*	72	8236,24	4,94	12	8	2980	58,62	unstable	100	-0,535
**14.2**	*Methanonatronarchaeum thermophilum*	73	8538,97	6,58	13	13	2980	40,62	unstable	113,56	-0,545
**15**	*Methanopyrus kandleri AV19*	73	8146,34	4,64	15	9	125	30,39	stable	122,6	0,052
**16**	*Haloarcula hispanica ATCC 33960*	60	6490,23	4,01	12	3	1490	21,24	stable	100,5	-0,123
**17**	*Halomicrobium mukohataei DSM 12286*	60	6542,31	4,3	12	4	2980	22,83	stable	102,17	-0,248
**18.1**	*Halorhabdus tiamatea SARL4B*	54	5779,42	3,94	12	2	1490	8,97	stable	129,81	0,183
**18.2**	*Halorhabdus tiamatea SARL4B*	61	6558,2	3,96	14	2	1490	19,45	stable	119,67	-0,033
**19**	*Halorientalis persicus*	61	6550,3	4,09	11	3	2980	32,61	stable	95,74	-0,193
**20**	*Halosimplex carlsbadense 2-9-1*	61	6661,34	4,05	13	4	2980	29,35	stable	98,85	-0,367
**21**	*Natronomonas pharaonis DSM 2160*	61	6656,37	4,19	12	3	2980	33,54	stable	98,85	-0,298
**22**	*Haladaptatus sp. R4*	60	6565,18	4,1	10	3	2980	29,05	stable	97,33	-0,192
**23**	*Halalkalicoccus jeotgali*	61	6574,3	3,92	12	3	1490	18,82	stable	103,61	-0,043
**24**	*Halanaeroarchaeum sulfurireducens*	63	6848,59	4,32	13	4	1490	35,28	stable	97,3	-0,267
**25**	*Halarchaeum acidiphilum MH1-52-1*	64	6875,69	4,16	12	3	1490	26,38	stable	110,94	-0,033
**26**	*Haloarchaeobius iranensis*	73	7938,76	4,46	13	7	2980	28,2	stable	82,6	-0,533
**27**	*Halobacterium salinarum NRC-1*	69	7586,45	4,27	14	6	2980	35,47	stable	85,94	-0,355

Code	Species name	Aa	MW (Da)	pI	Asp + Glu	Arg + Lys	Extinction coefficient (M^{-1} cm^{-1})	Instability index	Stability	Aliphatic index	GRAVY
**28**	*Halodesulfurarchaeum formicicum*	61	6520,3	4,01	12	3	1490	28,43	stable	108,52	0,115
**29**	*Halovenus aranensis*	64	6903,62	4,09	12	4	1490	22,08	stable	92,66	-0,266
**30**	*Natronoarchaeum philippinense*	75	8001,75	3,75	17	3	1490	26,57	stable	100	-0,216
**31**	*Halococcus thailandensis JCM 13552*	78	8042,74	3,98	15	4	2980	34,38	stable	89,87	-0,265
**32**	*Halobellus limi*	77	8177,11	3,83	15	3	4470	20,75	stable	106,23	0,103
**33**	*Haloferax mediterranei ATCC 33500*	76	8243,21	3,9	16	4	2980	38,32	stable	111,45	-0,029
**34**	*Haloferax volcanii DS2*	76	8257,24	3,9	16	4	2980	33,81	stable	111,45	-0,028
**35**	*Halogeometricum borinquense DSM 11551*	77	8280,18	3,98	16	4	4470	47,22	unstable	102,47	-0,101
**36**	*Halogranum gelatinilyticum*	77	8293,18	3,86	17	4	2980	19,74	stable	106,23	-0,061
**37**	*Halopelagius longus*	79	8529,45	3,92	17	4	4470	55,28	unstable	98,61	-0,211
**38**	*Haloprofundus marisrubri*	75	8105,85	3,78	16	3	2980	27,92	stable	98,53	-0,151
**39**	*Haloquadratum walsbyi DSM 16790*	77	8145,04	3,93	14	3	2980	22,94	stable	100	-0,006
**40**	*Halobaculum gomorrense*	164	17566,65	4,75	23	14	11460	29,32	stable	87,87	-0,268
**41**	*Halohasta litchfieldiae*	78	8151,97	3,8	15	3	2980	14,47	stable	102,31	0,013
**42**	*Halolamina pelagica*	76	8221,03	3,95	16	3	2980	35,36	stable	102,37	-0,114
**43**	*Halonotius sp. J07HN4*	81	8649,57	4,04	18	5	not be visible by UV spectrophotometry	28,61	stable	100,99	-0,095
**44**	*Halopenitus persicus*	78	8482,36	4,15	16	5	4470	36	stable	96,15	-0,2
**45**	*Halorubrum lacusprofundi ATCC 49239*	80	8791,72	3,98	19	5	4470	38,91	stable	102,25	-0,182
**46**	*Salinigranum rubrum*	76	8146,06	4,15	14	3	2980	14,91	stable	114,08	0,076

Code	Species name	Aa	MW (Da)	pI	Asp + Glu	Arg + Lys	Extinction coefficient (M^{-1} cm^{-1})	Instability index	Stability	Aliphatic index	GRAVY
**47**	*Halobiforma nitratireducens JCM 10879*	78	8387,22	4,12	17	6	2980	30,81	stable	96,15	-0,306
**48**	*Halopiger xanaduensis SH-6*	75	8128,04	4,09	16	5	2980	38,09	stable	99,87	-0,235
**49**	*Halostagnicola larsenii*	75	8146,95	3,97	17	5	2980	19,27	stable	101,2	-0,284
**50**	*Haloterrigena saccharevitans*	74	7967,87	4,08	15	5	2980	24,87	stable	102,57	-0,169
**51**	*Halovivax asiaticus JCM 14624*	99	10605,78	4,33	17	8	8480	27,97	stable	86,67	-0,283
**52**	*Natrialba asiatica*	78	8312,02	3,99	17	5	2980	18,22	stable	87,44	-0,376
**53**	*Natrinema versiforme*	75	8112,04	4,09	16	5	2980	33,13	stable	103,73	-0,171
**54**	*Natronobacterium gregoryi SP2*	75	8067,81	3,97	17	5	2980	26,65	stable	97,33	-0,264
**55**	*Natronococcus occultus SP4*	75	8246,05	4,12	18	7	2980	20,35	stable	94,67	-0,448
**56**	*Natronolimnobius baerhuensis*	81	8584,27	3,88	18	5	2980	29,77	stable	87,78	-0,368
**57**	*Natronorubrum sulfidifaciens*	75	8085,96	4,04	16	5	2980	32,53	stable	101,2	-0,208
**58**	*Salinarchaeum sp. Harcht-Bsk1*	88	9283,95	3,93	20	6	2980	26,8	stable	84,09	-0,423
**59.1**	*Methanocella conradii HZ254*	72	8141,28	5,02	12	9	2980	52,11	unstable	100	-0,408
**59.2**	*Methanocella conradii HZ254*	74	8171,55	8,06	8	9	1490	34,71	stable	119,73	-0,127
**60**	*Methanocalculus sp. 52_23*	78	8585,04	5,25	12	10	1490	30,33	stable	133,59	0,168
**61**	*Methanocorpusculum sp. MCE*	77	8449,83	5,24	10	8	1490	34,13	stable	122,6	0,13
**62**	*Methanoculleus thermophilus*	75	8323,66	4,9	11	8	2980	31,4	stable	125,87	-0,007

Code	Species name	Aa	MW (Da)	pI	Asp + Glu	Arg + Lys	Extinction coefficient (M^{-1} cm^{-1})	Instability index	Stability	Aliphatic index	GRAVY
**63.1**	*Methanofollis liminatans DSM 4140*	88	9694,27	5,02	13	9	4470	40,89	unstable	130,45	0,157
**63.2**	*Methanofollis liminatans DSM 4140*	78	8755,15	6,72	10	10	2980	10,04	unstable	122,18	-0,077
**64.1**	*Methanogenium bourgense*	75	8341,69	4,9	11	8	2980	31,97	stable	120,67	-0,041
**64.2**	*Methanogenium bourgense*	78	8640,06	6,72	10	10	1490	18,87	stable	122,18	0,017
**65.1**	*Methanolacinia petrolearia DSM 11571*	75	8325,63	5,7	9	8	2980	41,73	unstable	122	-0,119
**65.2**	*Methanolacinia petrolearia DSM 11571*	78	8753,24	5,61	12	10	2980	39,99	stable	126,15	0,004
**66.1**	*Methanoplanus limicola DSM 2279*	75	8244,56	5,73	10	9	2980	40,17	unstable	116,8	-0,089
**66.2**	*Methanoplanus limicola DSM 2279*	78	8683,04	6,06	11	10	2980	37,04	stable	121,15	-0,082
**67.1**	*Methanolinea sp. SDB*	78	8782,24	6,54	10	10	2980	38,66	stable	123,46	0,023
**67.2**	*Methanolinea sp. SDB*	75	8415,73	5,24	11	9	2980	43,33	unstable	118	-0,156
**68.1**	*Methanoregula boonei 6A8*	76	8413,78	5,69	10	9	2980	40,05	unstable	125,39	0,007
**68.2**	*Methanoregula boonei 6A8*	78	8699,26	6,56	10	10	1490	34,14	stable	134,74	0,271
**69.1**	*Methanosphaerula palustris E1-9c*	75	8189,39	4,87	11	8	2980	26,21	stable	115,47	-0,123
**69.2**	*Methanosphaerula palustris E1-9c*	77	8513,06	5,71	10	9	1490	32,68	stable	135,32	0,318
**70.1**	*Methanospirillum hungatei JF-1*	79	8699,08	5,29	11	9	2980	31,49	stable	120,76	0,076
**70.2**	*Methanospirillum hungatei JF-1*	80	8884,35	5,63	9	8	2980	42,69	unstable	115,62	-0,129
**71.1**	*Methanothrix soehngenii GP6*	56	6450,14	5,13	7	5	2980	45,9	unstable	135,54	0,196
**71.2**	*Methanothrix soehngenii GP6*	78	8622	5,24	11	9	2980	31,84	stable	111,03	-0,047

Code	Species name	Aa	MW (Da)	pI	Asp + Glu	Arg + Lys	Extinction coefficient (M^{-1} cm^{-1})	Instability index	Stability	Aliphatic index	GRAVY
**71.3**	*Methanothrix soehngenii GP6*	80	8802,15	9,1	9	11	2980	57,53	unstable	91,38	-0,277
**72.1**	*Methanococcoides burtonii*	72	7948,15	5,21	11	9	2680	21,8	stable	118,89	-0,022
**72.2**	*Methanococcoides burtonii*	65	7128,15	4,78	9	5	1490	49,37	unstable	124,31	0,003
**73.1**	*Methanohalobium evestigatum Z-7303*	72	8032,15	5,18	10	8	2980	30,6	stable	113,47	-0,194
**73.2**	*Methanohalobium evestigatum Z-7303*	74	8204,5	5,48	11	8	1490	37,43	stable	124,86	-0,023
**73.3**	*Methanohalobium evestigatum Z-7303*	105	12105,43	4,44	20	9	16055	43,71	unstable	89,05	-0,429
**74.1**	*Methanohalophilus mahii DSM 5219*	72	7977,2	5,71	10	9	2980	22,76	stable	116,25	-0,05
**74.2**	*Methanohalophilus mahii DSM 5219*	75	8480,82	6,06	11	10	1490	43,18	unstable	102,53	-0,307
**75.1**	*Methanolobus psychrophilus R15*	56	6058,96	4,73	8	4	1490	36,5	stable	132,14	0,23
**75.2**	*Methanolobus psychrophilus R16*	72	8030,3	6,55	10	10	2980	23,7	stable	116,25	-0,157
**76.1**	*Methanomethylovorans hollandica DSM 15978*	71	7975,14	5,73	10	9	2980	27,54	stable	108,31	-0,293
**76.2**	*Methanomethylovorans hollandica DSM 15978*	74	8234,52	5,83	10	8	1490	41,04	unstable	125	-0,057
**77.1**	*Methanosalsum zhilinae DSM 4017*	72	8030,26	5,69	10	9	2980	24,61	stable	117,64	-0,131
**77.2**	*Methanosalsum zhilinae DSM 4017*	102	11330,16	4,87	15	10	1490	43,15	unstable	125,98	0,093
**78**	*Methanosarcina acetivorans C2A*	74	8336,82	8,09	10	11	1490	28,22	stable	118,24	-0,05
**79**	*Methanosarcina mazei Go1*	74	8280,75	6,55	10	10	1490	26,81	stable	118,24	-0,028
**80.1**	*Palaeococcus pacificus DY20341*	78	8739,18	5,6	13	11	4470	27,09	stable	132,31	0,009

Code	Species name	Aa	MW (Da)	pI	Asp + Glu	Arg + Lys	Extinction coefficient (M^{-1} cm^{-1})	Instability index	Stability	Aliphatic index	GRAVY
**80.2**	*Palaeococcus pacificus DY20342*	73	8350,63	4,75	15	10	12490	41,94	unstable	107,95	-0,118
**81**	*Pyrococcus abyssi GE5*	75	8488,9	5,59	13	11	2980	33,8	stable	123,33	0,019
**82**	*Pyrococcus furiosus*	69	7782,02	4,55	14	7	11000	15,7	stable	119,86	0,072
**83**	*Thermococcus kodakarensis KOD1*	71	8160,39	4,65	13	9	13980	40,78	unstable	112,54	-0,142
**84.1**	*Acidilobus saccharovorans 345-15*	92	10196,81	7,96	11	12	5960	25,6	stable	110	-0,059
**84.2**	*Acidilobus saccharovorans 345-15*	149	16411,74	7,95	18	19	9970	33,48	stable	96,04	-0,099
**85.1**	*Caldisphaera lagunensis DSM 15908*	90	9879,58	7,94	11	12	4470	38,67	stable	104	0,004
**85.2**	*Caldisphaera lagunensis DSM 15908*	148	16688,01	7,86	18	19	17420	36,93	stable	92,84	-0,349
**85.3**	*Aeropyrum pernix K1*	77	8373,95	8,12	8	9	2980	43,79	stable	130,26	0,332
**86**	*Aeropyrum pernix K1*	94	10131,78	7,95	11	12	4470	10,36	stable	104,79	0,078
**87.1**	*Desulfurococcus amylolyticus DSM 16532*	75	8278,56	6,06	11	10	not be visible by UV spectrophotometry.	52,37	unstable	119,47	-0,181
**87.2**	*Desulfurococcus amylolyticus DSM 16532*	93	10098,63	8,79	9	11	7450	24,1	stable	96,24	-0,132
**87.3**	*Desulfurococcus amylolyticus DSM 16532*	149	17091,99	7,93	23	24	14440	38,34	stable	107,11	-0,203
**88.1**	*Ignicoccus hospitalis KIN4/1*	73	8097,49	6,07	12	11	not be visible by UV spectrophotometry.	41,31	unstable	114,66	0,062
**88.2**	*Ignicoccus hospitalis KIN4/1*	91	10321,03	7,82	12	13	7450	21,43	stable	101,65	-0,202
**89.1**	*Ignisphaera aggregans DSM 17230*	78	8637,04	6,83	10	10	6990	48,02	unstable	114,87	-0,092
**89.2**	*Ignisphaera aggregans DSM 17230*	94	10370,08	9,2	10	13	4470	32,64	stable	95,32	-0,082

Code	Species name	Aa	MW (Da)	pI	Asp + Glu	Arg + Lys	Extinction coefficient (M^{-1} cm^{-1})	Instability index	Stability	Aliphatic index	GRAVY
**89.3**	*Ignisphaera aggregans DSM 17230*	146	16103,52	7,92	19	20	8940	31,82	stable	111,37	-0,016
**90.1**	*Staphylothermus marinus F1*	75	8402,78	5,86	14	12	1490	19,77	stable	127,33	-0,187
**90.2**	*Staphylothermus marinus F1*	91	9954,65	9,1	7	10	7450	7,84	stable	104,84	0,03
**90.3**	*Staphylothermus marinus F1*	149	17010,84	7,9	22	23	8940	33,29	stable	111,21	-0,176
**91.1**	*Thermogladius calderae 1633*	78	8532,86	5,68	9	8	4470	15,44	stable	108,59	0,11
**91.2**	*Thermogladius calderae 1633*	150	17190,03	7,92	24	25	7450	35,9	stable	113,6	-0,248
**92.1**	*Thermosphaera aggregans DSM 11486*	76	8463,71	5	13	9	1490	62,99	unstable	115,26	-0,139
**92.2**	*Thermosphaera aggregans DSM 11486*	91	10105,77	9,48	7	12	8940	34,17	stable	98,46	-0,169
**92.3**	*Thermosphaera aggregans DSM 11486*	149	16757,61	6,92	22	22	12950	24,08	stable	112,35	-0,054
**93.1**	*Zestosphaera tikiterensis*	83	9331,82	8,03	10	11	4470	30,34	stable	112,53	-0,004
**93.2**	*Zestosphaera tikiterensis*	156	17255,02	7,89	18	19	8940	21,08	stable	116,15	0,074
**94.1**	*Hyperthermus butylicus DSM5456*	93	10214,04	9,46	10	14	4470	24,05	stable	106,56	0,083
**94.2**	*Hyperthermus butylicus DSM5456*	152	17079,9	9,22	20	23	10430	25,3	stable	108,36	-0,025
**95.1**	*Pyrodictium delaneyi*	96	10587,47	8,82	12	14	4470	23,56	stable	106,35	0,059
**95.2**	*Pyrodictium delaneyi*	148	16449,14	9,22	18	21	8940	32,67	stable	108,65	-0,012
**96**	*Pyrolobus fumarii 1A*	92	10288,99	6,26	13	13	5960	33,28	stable	104,67	-0,096
**97**	*Fervidicoccus fontis Kam940*	89	9987,54	8,69	10	12	7450	27,8	stable	90,9	-0,391
**98.1**	*Acidianus sulfidivorans*	87	9772,37	8,73	12	14	4470	28,97	stable	100,69	-0,329

Code	Species name	Aa	MW (Da)	pI	Asp + Glu	Arg + Lys	Extinction coefficient (M^{-1} cm^{-1})	Instability index	Stability	Aliphatic index	GRAVY
	JP7										
**98.2**	*Acidianus sulfidivorans JP7*	145	16414,85	6,33	19	19	8940	36,07	stable	111,52	-0,141
**99.1**	*Metallosphaera yellowstonensis MK1*	86	9702,3	7,82	12	13	4470	29,09	stable	103,02	-0,269
**99.2**	*Metallosphaera yellowstonensis MK1*	146	16306,71	8,83	18	20	7450	42,51	unstable	103,49	-0,214
**100.1**	*Saccharolobus solfataricus P2 (Sulfolobus)*	87	9788,33	7,83	12	13	4470	28,18	stable	101,84	-0,271
**100.2**	*Saccharolobus solfataricus P2 (Sulfolobus)*	146	16513,92	7,81	18	19	11920	31,64	stable	98,08	-0,366
**101.1**	*Sulfodiicoccus acidiphilus*	91	10057,63	7,82	12	13	4470	20,85	stable	102,64	-0,236
**101.2**	*Sulfodiicoccus acidiphilus*	149	16300,52	8,83	18	20	7450	33,29	stable	88,79	-0,279
**102.1**	*Sulfolobus acidocaldarius DSM 639*	79	8534,05	6,72	9	9	1490	26,77	stable	124,3	0,305
**102.2**	*Sulfolobus acidocaldarius DSM 639*	144	16026,47	7,83	18	19	8940	27,62	stable	103,4	-0,122
**103.1**	*Sulfurisphaera tokodaii str. 7*	90	10029,77	9,17	11	14	4470	25,71	stable	105	-0,12
**103.2**	*Sulfurisphaera tokodaii str. 7*	143	15996,27	7,81	18	19	13410	38,07	stable	106,29	-0,249
**104.1**	*Thermofilum pendens Hrk 5*	73	8102,35	6,57	10	10	2980	36,72	stable	117,4	-0,201
**104.2**	*Thermofilum pendens Hrk 5*	100	11304,77	4,95	16	12	1490	45,35	unstable	75	-0,621
**105.1**	*Caldivirga maquilingensis IC-167*	79	8799,25	6,54	10	10	1490	33,16	stable	103,42	0,011
**105.2**	*Caldivirga maquilingensis IC-167*	86	9734,39	8,93	10	12	2980	17,79	stable	114,19	-0,042
**105.3**	*Caldivirga maquilingensis IC-167*	140	15885,41	6,39	21	21	8940	39,45	stable	107,07	-0,114

Code	Species name	Aa	MW (Da)	pI	Asp + Glu	Arg + Lys	Extinction coefficient $(M^{-1} cm^{-1})$	Instability index	Stability	Aliphatic index	GRAVY
**106.1**	*Pyrobaculum aerophilum DSM 7523*	80	8869,23	5,54	11	9	1490	52,87	unstable	112	0,042
**106.2**	*Pyrobaculum aerophilum DSM 7524*	85	9188,86	9,72	7	13	4470	35,56	stable	105,41	-0,019
**106.3**	*Pyrobaculum aerophilum DSM 7525*	144	16362,89	7,94	20	21	7450	36,12	stable	101,46	-0,145
**107**	*Thermocladium sp. ECH_B*	139	15612,93	7,89	18	19	12950	34,9	stable	98,85	-0,253
**108.1**	*Thermoproteus tenax*	86	9448,19	9,61	8	13	4470	53,45	unstable	117,79	0,101
**108.2**	*Thermoproteus tenax*	140	15884,31	7,81	19	20	14900	49,76	unstable	105,07	-0,169
**109.1**	*Vulcanisaeta distribute DSM 14429*	77	8475,98	7,91	8	9	1490	46,6	unstable	117,53	0,043
**109.2**	*Vulcanisaeta distribute DSM 14429*	81	9253,81	7,86	11	12	4470	33,14	stable	110,49	-0,327
**109.3**	*Vulcanisaeta distribute DSM 14429*	140	15722,24	7,97	17	18	5960	40,51	unstable	100,14	0,041

Table S3. Gene environment of the *lsm* gene in the different genomes of species of the *Archaea* domain. The genes encoding the different proteins are represented in different colours corresponding to: Lsm protein, 50S ribosomal L37e protein, RNA-binding proteins, transcriptional factors, transcriptional regulators, metabolic enzymes, proteins related to DNA metabolism, transporters, tRNA guanosine transglycosylase, methionine adenosyltransferase, and hypothetical proteins or proteins related to other functions.

Species name	Gene environment	Encoded proteins
**Phylum Nanoarchaeota – Nanoarchaeales - Nanoarchaeaceae**		
**1.** ***Nanoarchaeum equitans*** **Kin4-M**	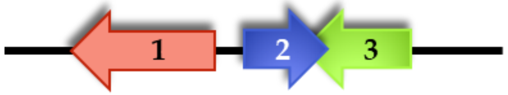	**1:** Transcription factor<br>**2: Putative U6 small nuclear ribonucleoprotein**<br>**3:** 50S ribosomal protein L37Ae
**Phylum Euryarchaeota**		

Species name	Gene environment	Encoded proteins
	**Archaeoglobi – Archaeoglobales - Archaeoglobaceae**	
**2.** ***Archaeglobus fulgidus*** **DSM 4304**	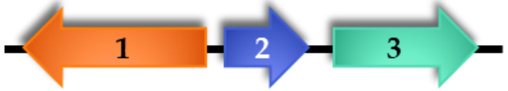	**1:** NAD-dependent epimerase/dehydratase family protein<br>**2: Like-Sm ribonucleoprotein core**<br>**3:** Lrp/AsnC family transcriptional regulator
**3.1.** ***Ferroglobus placidus DSM 10642***	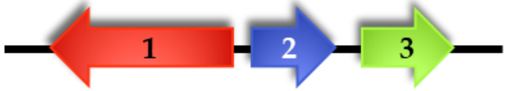	**1:** RNA-binding protein<br>**2: Like-Sm ribonucleoprotein core**<br>**3:** 50S ribosomal protein L37e
**3.2.** ***Ferroglobus placidus*** **DSM 10642**	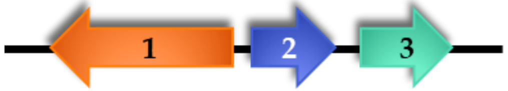	**1:** NAD-dependent epimerase/dehydratase family protein<br>**2: Like-Sm ribonucleoprotein core**<br>**3:** H-T-H transcriptional regulator
**4.1.** ***Geoglobus acetinovorans***	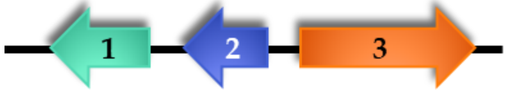	**1:** MarR family transcriptional regulator<br>**2: Like-Sm ribonucleoprotein core**<br>**3:** NAD-dependent epimerase/dehydratase family protein
**4.2.** ***Geoglobus acetinovorans***	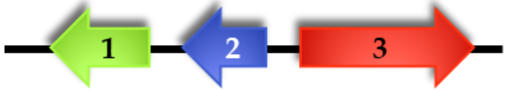	**1:** 50S ribosomal protein L37e<br>**2: Small nuclear ribonucleoprotein**<br>**3:** RNA-binding protein
	**Methanobacteria - Methanobacteriales - Methanobacteriaceae**	
**5.** ***Methanobacterium lacus***	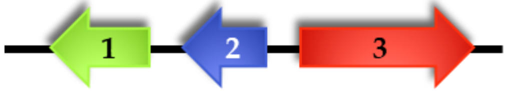	**1:** 50S ribosomal protein L37e<br>**2: Small nuclear ribonucleoprotein**<br>**3:** RNA-binding protein
**6.** ***Methanosphaera stadtmanae*** **DSM 3091**	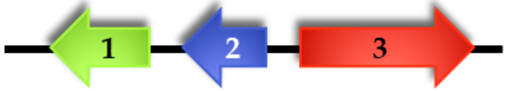	**1:** 50S ribosomal protein L37e<br>**2: Small nuclear ribonucleoprotein**<br>**3:** RNA-binding protein
	**Methanobacteria - Methanobacteriales -Methanothermaceae**	
**7.1** ***Methanothermus fervidus*** **DSM 2088**	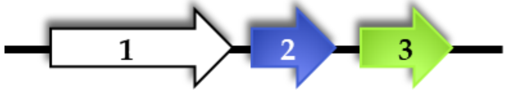	**1:** Hypothetical protein<br>**2: Small nuclear ribonucleoprotein**<br>3: 50S ribosomal protein L37e

**Species name**	**Gene environment**	**Encoded proteins**
**7.2 *Methanothermus fervidus* DSM 2088**	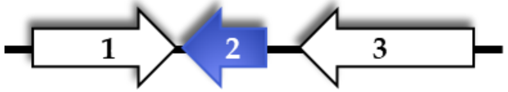	**1:** Methanogenesis marker 12 protein<br>**2: Like-Sm ribonucleoprotein core**<br>**3:** Methyltransferase
	**Methanococci – Methanococcales - Methanocaldococcaceae**	
**8. *Methanocaldococcus jannaschii***	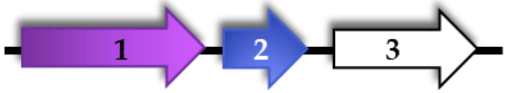	**1:** Endonuclease III domain-containing protein<br>**2: RNA chaperone Hfq**<br>**3:** Energy-converting dehydrogenase B subunit P
	**Methanococci – Methanococcales - Methanococcaceae**	
**9. *Methanococcus maripaludis* C5**	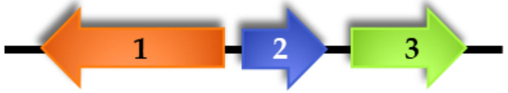	**1: 3-isopropylmalate dehydratase large subunit**<br>**2:** Small nuclear ribonucleoprotein<br>**3:** 50S ribosomal protein L37e
	**Methanopyri – Methanopyrales - Methanopyraceae**	
**10. *Methanopyrus kandleri* AV19**	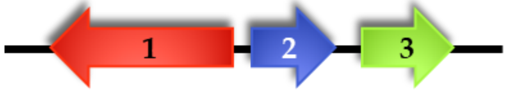	**1:** RNA-binding protein<br>**2: Like-Sm ribonucleoprotein core**<br>**3:** 50S ribosomal protein L37e
	**Halobacteria – Halobacteriales - Haloarculaceae**	
**11. *Haloarcula hispanica* ATCC 33960**	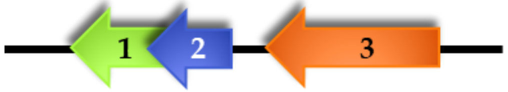	**1:** 50S ribosomal protein L37e<br>**2: Small nuclear ribonucleoprotein**<br>**3:** Metallo-hydrolase
**12. *Natronomonas pharaonis* DSM 2160**	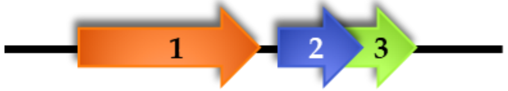	**1:** Metallo-hydrolase<br>**2: RNA-binding protein Lsm**<br>**3:** 50S ribosomal protein L37e
	**Halobacteria – Halobacteriales - Halobacteriaceae**	
**13. *Halanaeroarchaeum sulfurireducens***	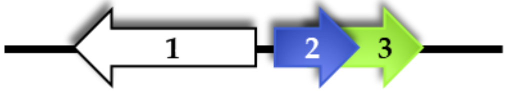	**1:** Zinc-dependent metalloprotease<br>**2: Like-Sm ribonucleoprotein core**<br>**3:** 50S ribosomal protein L37e
**14. *Halobacterium salinarum* NRC-1**	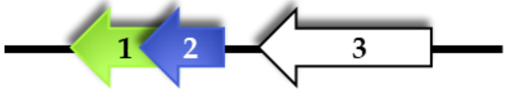	**1:** 50S ribosomal protein L37e<br>**2: Like-Sm ribonucleoprotein core**<br>**3:** Metalloprotease

Species name	Gene environment	Encoded proteins
	**Halobacteria –Haloferacales - Haloferacaceae**	
**15.** ***Haloferax mediterranei*** **ATCC 33500**	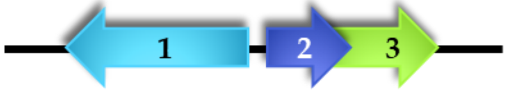	**1:** Ribonuclease J<br>**2: Like-Sm ribonucleoprotein core**<br>**3:** 50S ribosomal protein L37e
**16.** ***Halogeometricum borinquense*** **DSM 11551**	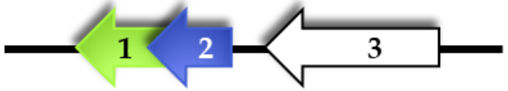	**1:** 50S ribosomal protein L37e<br>**2: Like-Sm ribonucleoprotein core**<br>**3:** Sensor histidine kinase
**17.** ***Haloquadratum walsbyi*** **DSM 16790**	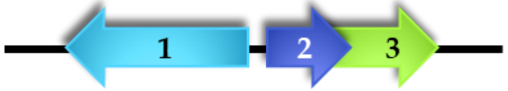	**1:** Ribonuclease J<br>**2: Like-Sm ribonucleoprotein core**<br>**3:** 50S ribosomal protein L37e
	**Halobacteria – Haloferacales - Halorubraceae**	
**18.** ***Halohasta litchfieldiae***	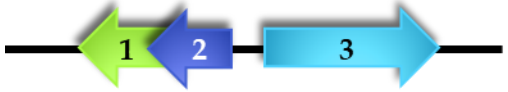	**1:** 50S ribosomal protein L37e<br>**2: Like-Sm ribonucleoprotein core**<br>**3:** Ribonuclease J
**19.** ***Halorubrum lacusprofundi*** **ATCC 49239**	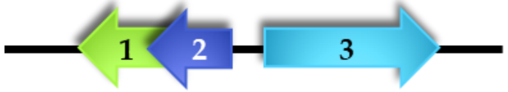	**1:** 50S ribosomal protein L37e<br>**2: Like-Sm ribonucleoprotein core**<br>**3:** Ribonuclease J
	**Halobacteria – Natrialbales - Natrialbaceae**	
**20.** ***Halopiger xanaduensis*** **SH-6**	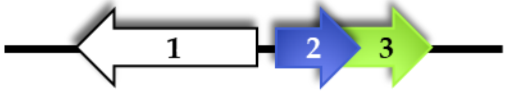	**1:** Hypothetical protein<br>**2: Like-Sm ribonucleoprotein core**<br>**3:** 50S ribosomal protein L37e
**21.** ***Natronobacterium gregoryi*** **SP2**	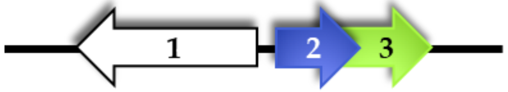	**1:** Hypothetical protein<br>**2: Like-Sm ribonucleoprotein core**<br>**3:** 50S ribosomal protein L37e
	**Methanomicrobia – Methanocellales – Methanocellaceae**	
**22.1** ***Methanocella conradii*** **HZ254**	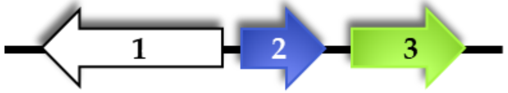	**1:** Hypothetical protein<br>**2: Like-Sm ribonucleoprotein core**<br>**3:** 50S ribosomal protein L37e

Species name	Gene environment	Encoded proteins
**22.2** ***Methanocella conradii*** **HZ254**	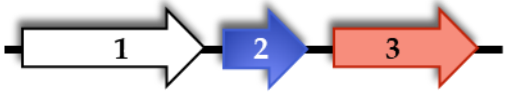	**1:** Flavodoxin domain-containing protein<br>**2: Like-Sm ribonucleoprotein core**<br>**3:** Transcription factor
	**Methanomicrobia - Methanomicrobiales - Methanomicrobiaceae**	
**23.1** ***Methanolacinia petrolearia*** **DSM 11571**	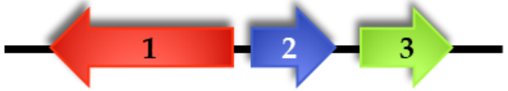	**1:** RNA-binding protein<br>**2: Like-Sm ribonucleoprotein core**<br>**3:** 50S ribosomal protein L37e
**23.2** ***Methanolacinia petrolearia*** **DSM 11571**	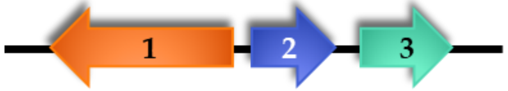	**1:** Fructose-1,6-bisphosphatase<br>**2: Like-Sm ribonucleoprotein core**<br>**3:** H-T-H transcriptional regulator
**24.1** ***Methanogenium bourgense***	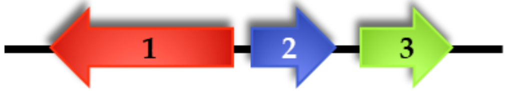	**1:** RNA-binding protein<br>**2: Like-Sm ribonucleoprotein core**<br>**3:** 50S ribosomal protein L37e
**24.2** ***Methanogenium bourgense***	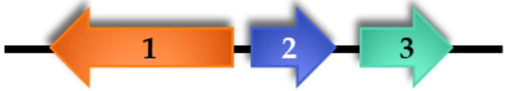	**1:** Fructose-1,6-bisphosphatase<br>**2: Like-Sm ribonucleoprotein core**<br>**3:** AsnC family transcriptional regulator
	**Methanomicrobia - Methanomicrobiales - Methanoregulaceae**	
**25.1** ***Methanoregula boonei*** **6A8**	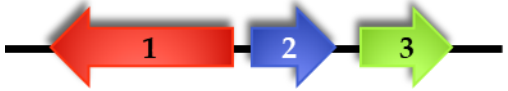	**1:** RNA-binding protein<br>**2: Like-Sm ribonucleoprotein core**<br>**3:** 50S ribosomal protein L37e
**25.2** ***Methanoregula boonei*** **6A8**	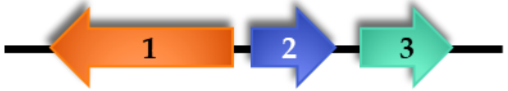	**1:** Fructose-1,6-bisphosphatase<br>**2: Like-Sm ribonucleoprotein core**<br>**3:** MarR family transcriptional regulator:
**26.1** ***Methanosphaerula palustris*** **E1-9c**	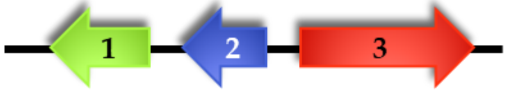	**1:** 50S ribosomal protein L37e<br>**2: Small nuclear ribonucleoprotein**<br>**3:** RNA-binding protein
**26.2** ***Methanosphaerula palustris*** **E1-9c**	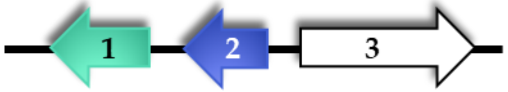	**1:** H-T-H transcriptional regulator:<br>**2: Small nuclear ribonucleoprotein**<br>**3:** Hypothetical protein

Species name	Gene environment	Encoded proteins
	**Methanomicrobia - Methanomicrobiales - Methanospirillaceae**	
**27. *Methanospirillum hungatei* JF-1**	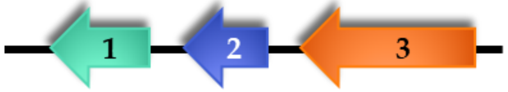	**1:** AsnC family transcriptional regulator<br>**2: Like-Sm ribonucleoprotein core**<br>**3:** Fructose-1,6-bisphosphatase
	**Methanomicrobia – Methanosarcinales - Methanosaetaceae**	
**28.1 *Methanothrix soehngenii* GP6**	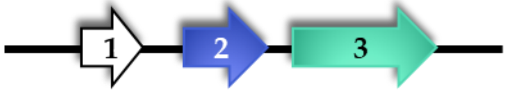	**1:** NifU family protein<br>**2: LSM domain-containing protein**<br>**3:** MarR family transcriptional regulator
**28.2 *Methanothrix soehngenii* GP6**	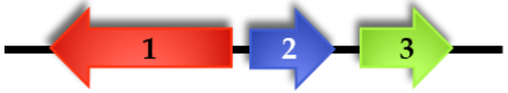	**1:** RNA-binding protein<br>**2: Like-Sm ribonucleoprotein core**<br>**3:** 50S ribosomal protein L37e
**28.3 *Methanothrix soehngenii* GP6**	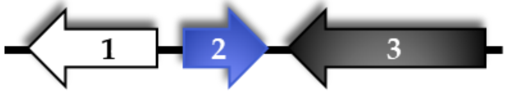	**1:** Hypothetical protein<br>**2: RNA chaperone Hfq**<br>**3:** Sodium:solute symporter family protein
	**Methanomicrobia – Methanosarcinales - Methanosarcinaceae**	
**29.1 *Methanococcoides burtonii***	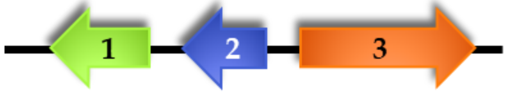	**1:** 50S ribosomal protein L37e<br>**2:** Like-Sm ribonucleoprotein<br>**3:** Bifunctional hydroxymethylpyrimidine kinase/phosphomethylpyrimidine kinase
**29.2 *Methanococcoides burtonii***	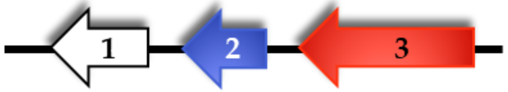	**1:** Hypothetical protein<br>**2: LSM domain-containing protein**<br>**3:** RNA-binding protein
**30.1 *Methanohalobium evestigatum* Z-7303**	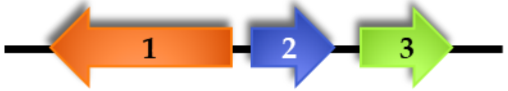	**1:** Bifunctional hydroxymethylpyrimidine kinase/phosphomethylpyrimidine kinase<br>**2: Like-Sm ribonucleoprotein**<br>**3:** 50S ribosomal protein L37e
**30.2 *Methanohalobium evestigatum* Z-7303**	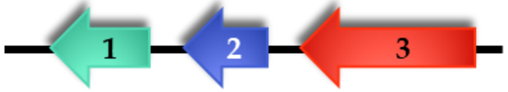	**1:** MarR family transcriptional regulator:<br>**2: Like-Sm ribonucleoprotein**<br>**3:** RNA-binding protein

Species name	Gene environment	Encoded proteins
**31.** ***Methanosarcina acetivorans*** **C2A**	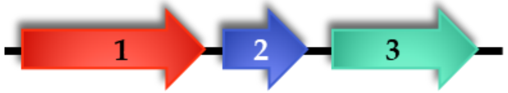	**1:** RNA-binding protein<br>**2: Like-Sm ribonucleoprotein**<br>**3:** Lrp/AsnC family transcriptional regulator
**32.** ***Methanosarcina mazei*** **Go1**	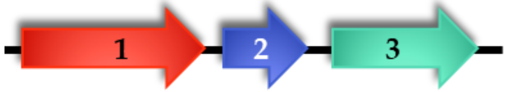	**1:** RNA-binding protein<br>**2: Like-Sm ribonucleoprotein**<br>**3:** Lrp/AsnC family transcriptional regulator
	**Thermococci –Thermococcales - Thermococcaceae**	
**33.1** ***Palaeococcus pacificus*** **DY20341**	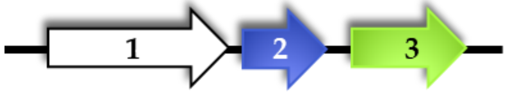	**1:** Hypothetical protein<br>**2: Like-Sm ribonucleoprotein**<br>**3:** 50S ribosomal protein L37e
**33.2** ***Palaeococcus pacificus*** **DY20341**	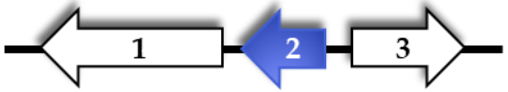	**1:** Hypothetical protein<br>**2: Like-Sm ribonucleoprotein**<br>**3:** Hypothetical protein
**34.** ***Pyrococcus abyssi*** **GE5**	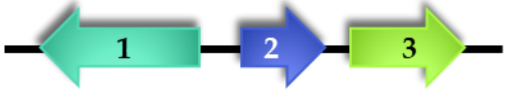	**1:** Lrp/AsnC family transcriptional regulator<br>**2: Like-Sm ribonucleoprotein**<br>**3:** 50S ribosomal protein L37e
**35.** ***Thermococcus kodakarensis*** **KOD1**	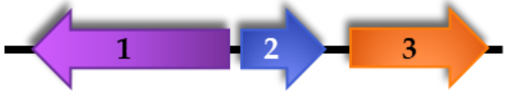	**1:** Transposase<br>**2: Like-Sm ribonucleoprotein**<br>**3:** Molybdopterin-guanine dinucleotide biosynthesis protein B
	**Phylum Crenarchaeota**	
	**Thermoprotei - Acidilobales - Acidilobaceae**	
**36.1** ***Acidilobus saccharovorans*** **345-15**	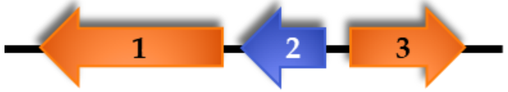	**1:** Myo-inositol-1-phosphate synthase<br>**2: Sm-like protein LSm6**<br>**3:** Fumarylacetoacetate hydrolase family protein
**36.2** ***Acidilobus saccharovorans*** **345-15**	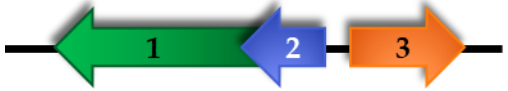	**1:** tRNA guanosine(15) transglycosylase TgtA<br>**2: Lsm family RNA-binding protein**<br>**3:** Acyl-CoA thioesterase
	**Thermoprotei- Acidilobales - Caldisphaeraceae**	

Species name	Gene environment	Encoded proteins
**37.1 *Caldisphaera lagunensis* DSM 15908**	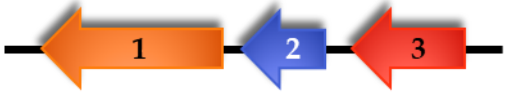	**1:** Myo-inositol-1-phosphate synthase<br>**2: Sm-like protein LSm6**<br>**3:** RNA-binding protein
**37.2 *Caldisphaera lagunensis* DSM 15908**	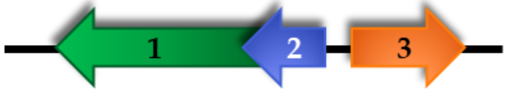	**1:** tRNA guanosine(15) transglycosylase TgtA<br>**2: Lsm family RNA-binding protein**<br>**3:** Acyl-CoA thioesterase
	**Thermoprotei- Desulfurococcales - Desulfurococcaceae**	
**38.1 *Aeropyrum pernix* K1**	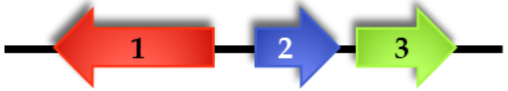	**1:** RNA-binding protein<br>**2: Small nucleolar RNP protein Sm**<br>**3:** 50S ribosomal protein L37e
**38.2 *Aeropyrum pernix* K1**	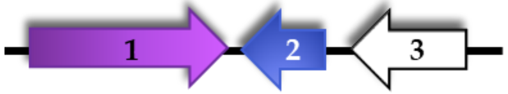	**1:** Endonuclease NucS<br>**2: Putative small nucleolar RNP protein Sm**<br>**3:** Hypothetical protein
**39.1 *Desulfurococcus amylolyticus* DSM 16532**	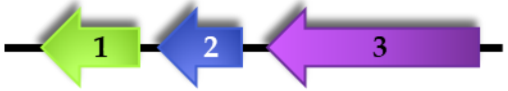	**1:** 50S ribosomal protein L37e<br>**2: Like-Sm ribonucleoprotein core**<br>**3:** DNA polymerase II
**39.2 *Desulfurococcus amylolyticus* DSM 16532**	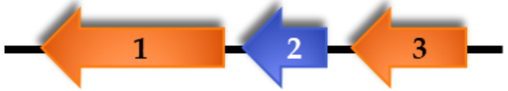	**1:** MBL fold metallo-hydrolase<br>**2: Small nuclear ribonucleoprotein, LSM family**<br>**3:** Class I SAM-dependent methyltransferase
**39.3 *Desulfurococcus amylolyticus* DSM 16532**	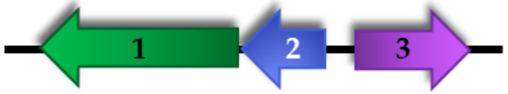	**1:** tRNA guanosine(15) transglycosylase TgtA<br>**2: Lsm family RNA-binding protein**<br>**3:** DNA-directed RNA polymerase subunit G
**40.1 *Thermosphaera aggregans* DSM 11486**	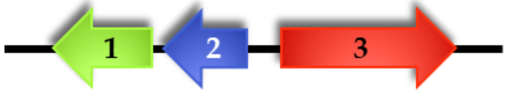	**1:** 50S ribosomal protein L37e<br>**2: Lsm family small nuclear ribonucleoprotein**<br>**3:** RNA-binding protein
**40.2 *Thermosphaera aggregans* DSM 11486**	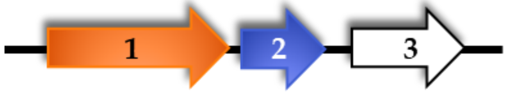	**1:** Class I SAM-dependent methyltransferase<br>**2: Lsm family small nuclear ribonucleoprotein**<br>**3:** Hypothetical protein

Species name	Gene environment	Encoded proteins
**40.3** ***Thermosphaera aggregans*** **DSM 11486**	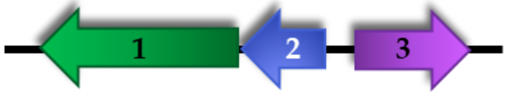	**1:** tRNA guanosine(15) transglycosylase TgtA<br>**2: Lsm family RNA-binding protein**<br>**3:** DNA-directed RNA polymerase subunit G
	**Thermoprotei - Desulfurococcales – Pyrodictiaceae**	
**41.1** ***Hyperthermus butylicus*** **DSM5456**	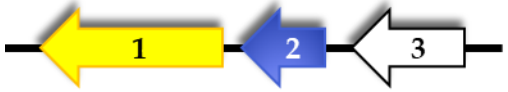	**1:** Methionine adenosyltransferase<br>**2: Sm-like protein LSm6**<br>**3:** Hypothetical protein
**41.2** ***Hyperthermus butylicus*** **DSM5456**	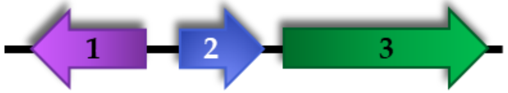	**1:** DNA-directed RNA polymerase subunit G<br>**2: Lsm family RNA-binding protein**<br>**3:** tRNA guanosine(15) transglycosylase TgtA
**42.1** ***Pyrodictium delaneyi***	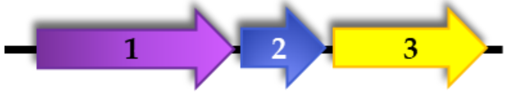	**1:** AbrB/MazE/SpoVT family DNA-binding domain-containing protein<br>**2: Sm-like protein LSm6**<br>**3:** Methionine adenosyltransferase
**42.2** ***Pyrodictium delaneyi***	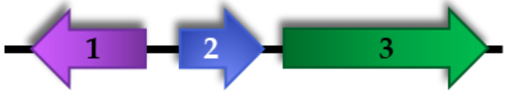	**1:** DNA-directed RNA polymerase subunit G<br>**2: Lsm family RNA-binding protein**<br>**3:** tRNA guanosine(15) transglycosylase TgtA
	**Thermoprotei – Fervidicoccales - Fervidicoccaceae**	
**43.** ***Fervidicoccus fontis*** **Kam940**	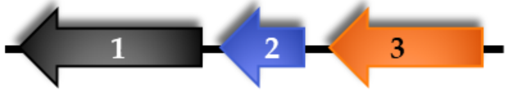	**1:** Mechanosensitive ion channel<br>**2: Like-Sm ribonucleoprotein core**<br>**3:** MBL fold metallo-hydrolase
	**Thermoprotei - Sulfolobales – Sulfolobaceae**	
**44.1** ***Acidianus sulfidivorans*** **JP7**	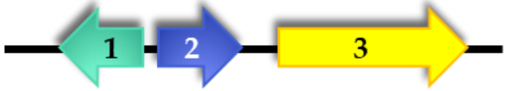	**1:** H-T-H transcriptional regulator<br>**2: Sm-like protein**<br>**3:** Methionine adenosyltransferase
**44.2** ***Acidianus sulfidivorans*** **JP7**	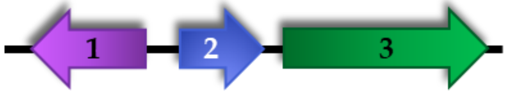	**1:** DNA-directed RNA polymerase subunit G<br>**2: Lsm family RNA-binding protein**<br>**3:** tRNA guanosine(15) transglycosylase TgtA

**Species name**	**Gene environment**	**Encoded proteins**
**45.1** ***Sulfolobus acidocaldarius*** **DSM 639**	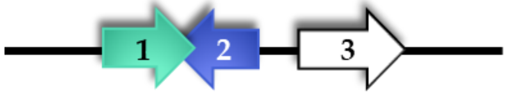	**1:** MarR family transcriptional regulator:<br>**2: Like-Sm ribonucleoprotein**<br>**3:** Hypothetical protein
**45.2** ***Sulfolobus acidocaldarius*** **DSM 639**	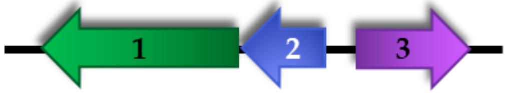	**1:** tRNA guanosine(15) transglycosylase TgtA<br>**2: Lsm family RNA-binding protein**<br>**3:** DNA-directed RNA polymerase subunit G
	**Thermoprotei - Thermoproteales - Thermofilaceae**	
**46.1** ***Thermofilum pendens*** **Hrk 5**	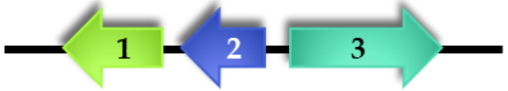	**1:** 50S ribosomal protein L37e<br>**2: Like-Sm ribonucleoprotein, core**<br>**3:** H-T-H transcriptional regulator
**46.2** ***Thermofilum pendens*** **Hrk 5**	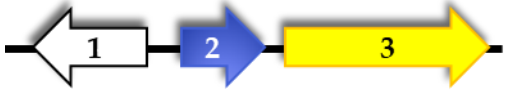	**1:** Hypothetical protein<br>**2: Like-Sm ribonucleoprotein, core**<br>**3:** Methionine adenosyltransferase
	**Thermoprotei - Thermoproteales - Thermoproteaceae**	
**47.1** ***Caldivirga maquilingensis*** **IC-167**	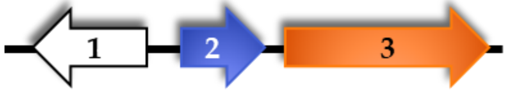	**1:** Hypothetical protein<br>**2: small nuclear ribonucleoprotein (Sm)**<br>**3:** Diphthine--ammonia ligase
**47.2** ***Caldivirga maquilingensis*** **IC-167**	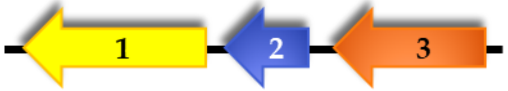	**1:** Methionine adenosyltransferase<br>**2: Like-Sm ribonucleoprotein core**<br>**3:** NCS2 family permease
**47.3** ***Caldivirga maquilingensis*** **IC-167**	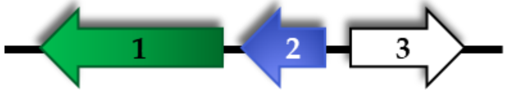	**1:** tRNA guanosine(15) transglycosylase TgtA<br>**2: Lsm family RNA-binding protein**<br>**3:** Hypothetical protein
**48.1** ***Thermoproteus tenax***	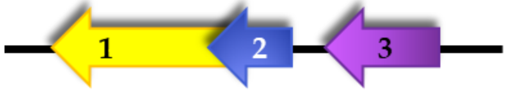	**1:** Methionine adenosyltransferase<br>**2: small nuclear ribonucleoprotein (Sm)**<br>**3:** DNA-directed RNA polymerase subunit N
**48.2** ***Thermoproteus tenax***	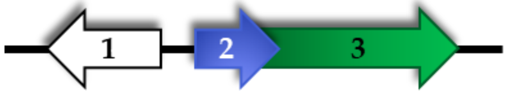	**1:** Hypothetical protein<br>**2: Lsm family RNA-binding protein**<br>**3:** tRNA guanosine(15) transglycosylase TgtA

Table S4. Overlap analysis of *lsm* and *rpl37e* genes in 80 species of the phylum Euryarchaeota.

Species name	Overlap	Overlapping nucleotides	Adjacent	Distance (ntds)
**Archaeoglobi – Archaeoglobales - Archaeoglobaceae**				
Archaeoglobus sulfaticallidus PM70-1	NO	**-**	**YES**	20
Archaeoglobus fulgidus DSM 4304	**YES**	1	NO	-
Archaeoglobus profundus DSM 5631	NO	-	**YES**	6
Ferroglobus placidus DSM 10642	NO	-	**YES**	9
Halobacteria – Halobacteriales - Haloarculaceae				
Haloarcula hispanica ATCC 33960	**YES**	4	NO	-
Haloarcula marismortui ATCC 43049	**YES**	4	NO	-
Halomicrobium mukohataei DSM 12286	**YES**	4	NO	-
Halorhabdus tiamatea SARL4B	**YES**	4	NO	-
Halorhabdus utahenyes DSM 12940	**YES**	4	NO	-
Natronomonas pharaonis DSM 2160	**YES**	4	NO	-
Halobacteria – Halobacteriales - Halobacteriaceae				
Halalkalicoccus jeotgali B3	**YES**	4	NO	-
Halobacterium_salinarum R1	**YES**	4	NO	-
Haloquadratum walsbyi C23	**YES**	4	NO	-
Haloquadratum walsbyi DSM 16854	**YES**	4	NO	-
Halobacteria – Halobacteriales - Halococcaceae				
Haloterrigena turkmenica DSM 5511	**YES**	4	NO	-
Halobacteria – Haloferacales - Haloferacaceae				
Haloferax alexandrinus JCM 10717	**YES**	4	NO	-
Haloferax denitrificans ATCC 35960	**YES**	4	NO	-
Haloferax elongansATCC BAA-1513	**YES**	4	NO	-
Haloferax gibbonSIiATCC 33959	**YES**	4	NO	-
Haloferax larseniiJCM 13917	**YES**	4	NO	-
Haloferax lucentenseDSM 14919	**YES**	4	NO	-
Haloferax mucosum ATCC BAA-1512	**YES**	4	NO	-
Haloferax prahovense DSM 18310	**YES**	4	NO	-
Haloferax sp. BAB2207	**YES**	4	NO	-
Haloferax sp. SB29	**YES**	4	NO	-
Haloferax sulfurifontis ATCC BAA-897	**YES**	4	NO	-
Haloferax volcanii DS2	**YES**	4	NO	-
Halogeometricum borinquense DSM 11551	**YES**	4	NO	-
Haloplanus natans DSM 17983	**YES**	4	NO	-
Halobacteria – Haloferacales - Halorubraceae				
Halolamina sp. IARI-CDK2	**YES**	4	NO	-
Halolamina sediminis strain halo7	**YES**	4	NO	-
Halopenitus sp. DYS4	**YES**	4	NO	-
Halorubrum aidingense JCM 13560	**YES**	4	NO	-
Halorubrum arcis JCM 13916	**YES**	4	NO	-
Halorubrum coriense DSM 10284	**YES**	4	NO	-
Halorubrum distributum JCM 9100	**YES**	4	NO	-
Halorubrum halophilumstrain B8	**YES**	4	NO	-
Halorubrum kocuriiJCM 14978	**YES**	4	NO	-
Halorubrum lacusprofundi ATCC49239	**YES**	4	NO	-
Halorubrum lipolyticum DSM 21995	**YES**	4	NO	-
Halorubrum litoreum JCM 13561	**YES**	4	NO	-
Halorubrum saccharovorum DSM 1137	**YES**	4	NO	-
Halorubrum sp. 5	**YES**	4	NO	-
Halorubrum sp. BV1	**YES**	4	NO	-

Species name	Overlap	Overlapping nucleotides	Adjacent	Distance (ntds)
Halorubrum sp. T3	**YES**	4	NO	-
Halorubrum tebenquichense DSM 14210	**YES**	4	NO	-
Halorubrum terrestre JCM 10247	**YES**	4	NO	-
Halobacteria – Natrialbales - Natrialbaceae				
Halobiforma lacisalYES AJ5	**YES**	4	NO	-
Halobiforma nitratireducens JCM 10879	**YES**	4	NO	-
Halopiger sp. IIH2	**YES**	4	NO	-
Halopiger xanaduensis SH-6	**YES**	4	NO	-
Haloterrigena turkmenica DSM 5511	**YES**	4	NO	-
Natrialba aegyptia DSM 13077	**YES**	4	NO	-
Natrialba asiatica DSM 12278	**YES**	4	NO	-
Natrialba hulunbeirensis JCM 10989	**YES**	4	NO	-
Natrinema sp. J7-2	**YES**	4	NO	-
Natronobacterium gregoryi SP2	**YES**	4	NO	-
Methanobacteria – Methanobacteriales - Methanobacteriaceae				
Methanobrevibacter ruminantium M1	NO	-	NO	-
Methanobrevibacter millerae	NO	-	NO	-
Methanobrevibacter sp. AbM4	NO	-	**YES**	112
Methanobrevibacter smithii ATCC 35061	NO	-	**YES**	58
Methanococci – Methanococcales - Methanococcaceae				
Methanococcus maripaludis OS7	NO	-	**YES**	76
Methanococcus vannielii SB	NO	-	**YES**	133
Methanomicrobia – Methanocellales - Methanocellaceae				
Methanocella paludicola SANAE	NO	-	**YES**	56
Methanomicrobia – Methanomicrobiales - Methanomicrobiaceae				
Methanoculleus marisnigri JR1	NO	-	**YES**	17
Methanomicrobia – Methanosarcinales - Methanosaetaceae				
Methanosaeta thermophila PT	NO	-	NO	-
Methanomicrobia – Methanosarcinales - Methanosarcinaceae				
Methanococcoides burtonii DSM 6242	NO	-	**YES**	495
Methanosarcina acetivorans C2A	NO	-	**YES**	143
Methanosarcina barkeri fusaro	NO	-	**YES**	150
Methanosarcina mazei Goe1	NO	-	**YES**	141
Methanospirillum hungatei JF-1	NO	-	**YES**	13
Thermococci – Thermococcales - Thermococcaceae				
Pyrococcus abyssi GE5	NO	-	**YES**	11
Pyrococcus furiosus DSM 3638	NO	-	**YES**	11
Pyrococcus horikoshii OT3	NO	-	**YES**	11
Thermococcus gammatolerans EJ3	NO	-	**YES**	25
Thermococcus kodakaraensis KOD1	NO	-	**YES**	20
Thermococcus onnurineus NA1	NO	-	**YES**	24
Thermococcus sibiricus MM 739	NO	-	**YES**	17
Thermoplasmata – Thermoplasmatales - Thermoplasmataceae				
Thermoplasma acidophilum DSM 1728	**YES**	4	NO	-
Thermoplasma volcanium GSS1	**YES**	4	NO	-
